# Determinants of divergent *Salmonella* and *Shigella* epithelial colonization strategies resolved in human enteroids and colonoids

**DOI:** 10.1128/mbio.00911-25

**Published:** 2025-05-30

**Authors:** Petra Geiser, Maria Letizia Di Martino, Ana C. C. Lopes, Alexandra Bergholtz, Magnus Sundbom, Martin Skogar, Wilhelm Graf, Kajsa Björner, Johan Vessby, Dominic-Luc Webb, Per M. Hellström, Jens Eriksson, Mikael E. Sellin

**Affiliations:** 1 Department of Medical Biochemistry and Microbiology, Uppsala University 211745, Uppsala, Uppsala County, Sweden; 2 Department of Surgical Sciences, Uppsala University 174469, Uppsala, Uppsala County, Sweden; 3 Department of Medical Sciences, Uppsala University 214437, Uppsala, Uppsala County, Sweden; 4 DLW Bioanalytics, Märsta, Sweden; 5 Science for Life Laboratory 463758, Uppsala, Uppsala County, Sweden; University of Washington, Seattle, Washington, USA

**Keywords:** *Salmonella*, *Shigella*, enteroids, colonoids, invasion, inflammasome, cell death

## Abstract

Despite their relatedness, *Salmonella* and *Shigella* enteropathogens differ in infectious dose, pathogenesis, and disease kinetics. The prototype strains *Salmonella enterica* serovar Typhimurium (*Salmonella*) and *Shigella flexneri* (*Shigella*) use Type-3-secretion-systems (T3SSs) to colonize intestinal epithelial cells (IECs). However, they have evolved unique sets of T3SS effectors and accessory virulence factors. A synthesis of how these differences impact the temporal progression of infection in non-transformed human epithelia is missing. Here, we followed *Salmonella* and *Shigella* infections of human enteroids and colonoids by time-lapse imaging to pinpoint virulence factor modules that shape the divergent epithelial colonization strategies. By an apical targeting module that integrates flagella and the SPI-4-encoded adhesin system with T3SS-1, *Salmonella* accomplishes appreciable numbers of apical IEC invasion events. These are promptly counteracted by IEC death, thus fostering a polyclonal iterative epithelial colonization strategy. The lack of a corresponding apical targeting module in *Shigella* makes this pathogen reliant on external factors, for example, preexisting damage, for rare apical access to the intraepithelial environment. However, *Shigella* compensates for this ineptness by an intraepithelial expansion module reliant on the tight coupling of OspC3-dependent temporal delay of cell death and IcsA-mediated mobility. This module enables intraepithelial *Shigella* to evade the IEC death response just long enough to expand laterally, thus fostering an essentially monoclonal colonization strategy. Taken together, the study reveals how a small set of virulence factors shapes divergent epithelial colonization strategies by related enterobacteria.

**IMPORTANCE:**

Pathogenic bacteria employ partially overlapping sets of virulence factor functions to colonize host epithelia but can differ markedly in their pathogenesis and disease kinetics. This work combines bacterial genetics and real-time microscopy in enteroids and colonoids to decipher the divergent colonization strategies of *Salmonella enterica* Typhimurium and *Shigella flexneri*—two major enteropathogens—in non-transformed human intestinal epithelia. The results reveal how virulence factor modules enabling efficiency at either the invasion step (*Salmonella*) or the intraepithelial expansion stage (*Shigella*) drive the radically different infection cycles of these related enterobacteria. Moreover, our work emphasizes how real-time studies in infection models that retain primary host cell features are critical to understanding infectious disease progression.

## INTRODUCTION

*Salmonella* and *Shigella* enteropathogens colonize the intestinal lumen and invade the epithelium. Despite their relatedness and a number of shared virulence traits, they exhibit important differences in their pathogenesis. Non-typhoidal *Salmonellae* display a broad host range and a variable infectious dose and primarily infect the distal small intestine and proximal colon in humans or the cecum in mice (1). *Shigellae* are primate-restricted with a very low infectious dose and selectively colonize the colonic epithelium *in vivo* (2, 3). Both *Salmonellae* and *Shigellae*, such as the commonly used prototype strains *Salmonella enterica* serovar Typhimurium and *Shigella flexneri* (hereafter referred to as *Salmonella* and *Shigella*, respectively), compete with the resident gut microbiota and face the soluble mucus layer loaded with antimicrobials as well as the glycocalyx composed of transmembrane mucins and other glycoproteins attached to the apical intestinal epithelial cell (IEC) surface (3, 4).

*Salmonella* employs flagellar motility to reach the epithelial surface (5–7), and flagella have also been implicated in binding to the host cell membrane (8, 9). An extensive repertoire of fimbrial and non-fimbrial adhesins (e.g., references [10–14]) can mediate initial *Salmonella* adhesion to host cells, as can the translocon mounted at the tip of the Type-3-secretion-system-1 (T3SS-1) (15, 16). *Shigella*, on the other hand, is non-flagellated, and only a few putative adhesins have been described (17–19). Their role during the invasion is more controversial, as alternative adhesion mechanisms have also been proposed (20). Both *Salmonella* and *Shigella* employ their syringe-like T3SS to deliver effectors that trigger bacterial uptake by the host cell (3, 21). While it is generally accepted that *Shigella* invades more efficiently from the basolateral side of IECs (22–24), several mechanisms have been proposed for how the epithelial barrier is penetrated from the apical side by either pathogen (20, 25–29). Subsequently, the T3SS is used to shape and maintain the bacteria’s intracellular niche within IECs. *Salmonella* predominantly coopts the endosomal pathway to establish a specialized *Salmonella*-containing vacuole (SCV) via the action of a second T3SS encoded by *Salmonella* pathogenicity island-2 (SPI-2) (30). By contrast, *Shigella* quickly escapes the endocytic vacuole and adopts a cytosolic lifestyle (3). The T3SS and its effectors are also involved in vacuolar escape (31), mobilization of host actin for cell-to-cell spread via the actin nucleator IcsA (32–34), and have been linked to suppression of host innate immune signaling (35–41).

One key epithelial innate immune mechanism comprises inflammasomes, cytosolic multiprotein complexes assembled upon detection of pathogen-associated molecular patterns (PAMPs) (42). A range of inflammasomes is activated by different PAMPs, such as flagellin, the T3SS rod and needle proteins, and lipopolysaccharide (43–45). Downstream of PAMP detection, they all converge at the cleavage of inflammatory caspases (Caspase-1, 4, 5, or 11), which results in prompt death and extrusion of infected IECs to restrict intraepithelial pathogen loads (46–53), along with proinflammatory cytokine secretion (49, 51, 53). Enteropathogens, on the other hand, have developed strategies to inhibit epithelial innate immunity. For *Shigella*, a number of effectors, such as OspC3 (35, 36, 54, 55), IpaH7.8 (40), and IpaH9.8 (37–39, 55), appear capable of suppressing inflammasome-signaling cascades at multiple levels.

Although a myriad of host cell interaction mechanisms have been documented for both *Salmonella* and *Shigella*, the synthesis of the decisive virulence factor modules and host cell responses dictating infection outcome remains to be fully established. The mechanisms and molecular targets of various virulence factors have been studied extensively in transformed or immortalized cell lines, and key phenotypes for effector mutants have been verified in animal models. However, cell lines incompletely recapitulate primary cell behavior, particularly when it comes to primary IEC-like architecture and functional cell death responses, and they appear hypersusceptible to enterobacterial colonization (29, 56). Furthermore, translating results to/from a highly complex animal model is far from trivial. Due to its primate-restricted nature, the pursuit of a reliable animal model for shigellosis has been challenging (57), and a mouse model reproducing some of the typical human pathogenesis has only been developed recently (46).

Enteroids and colonoids, derived from stem cells of the small and large intestine, respectively, offer non-transformed physiologically meaningful models of the gut epithelium. Experiments in enteroids/colonoids have proven capable of capturing gut bacterial virulence mechanisms and host responses at high resolution in both 2-dimensional (2D) and 3D-arranged intact mammalian epithelia (23, 24, 47, 48, 56, 58–64). Of note, these intestinal epithelial organoid models are only beginning to be explored for live-cell analyses. Hence, we have an incomplete understanding of how bacterial virulence factors and primary IEC responses interact dynamically across infection stages, and how this interplay differs between clinically significant bacteria. Toward this aim, we followed human enteroid and colonoid infections using time-lapse imaging to pinpoint virulence effector modules that shape divergent *Salmonella* and *Shigella* epithelial colonization strategies. Flagellar motility and the SPI-4-encoded adhesin system, together with T3SS-1, form an efficient apical targeting module for *Salmonella*. This results in numerous IEC invasion events, typically abrogated by the prompt induction of cell death to generate iterative cycles of IEC invasion and extrusion. *Shigella* lacks a corresponding apical targeting module but instead employs an efficient intraepithelial expansion module to compensate for the exceptionally rare apical IEC binding and invasion. Here, we found that the concerted actions of the inflammasome-targeting T3SS effector OspC3 and the actin nucleator IcsA allow *Shigella* to laterally outrun the epithelial cell death response just in time, thereby fostering an essentially monoclonal epithelial colonization strategy.

## RESULTS

### Unlike wild type or non-flagellated *Salmonella*, *Shigella* requires preexisting epithelial damage for apical invasion in microinjected human enteroids and colonoids

To follow *Salmonella* and *Shigella* colonization of intestinal epithelia in real time, we used comparative 3D jejunal enteroid and colonoid microinjections combined with time-lapse microscopy. It is well established that intraepithelial *Salmonella* predominantly occupies a vacuolar niche, the SCV (65), whereas *Shigella* swiftly escapes the endocytic compartment to colonize the host cell cytosol (3). These differences in intracellular lifestyle led us to use a vacuolar, SPI-2-driven reporter for *Salmonella* (p*ssaG*-GFP [50, 66]) but a glucose-6-phosphate transporter-based cytosolic reporter (p*uhpT*-GFP, informed by reference 67) for *Shigella*. While both the p*ssaG*-GFP and p*uhpT*-GFP reporters have been previously applied for *Salmonella* infections in human enteroids (62), the p*uhpT*-GFP reporter was verified for *Shigella* in Caco-2 cells and benchmarked against a constitutive reporter in enteroid-derived monolayers (Fig S1A and B).

**Fig 1:**
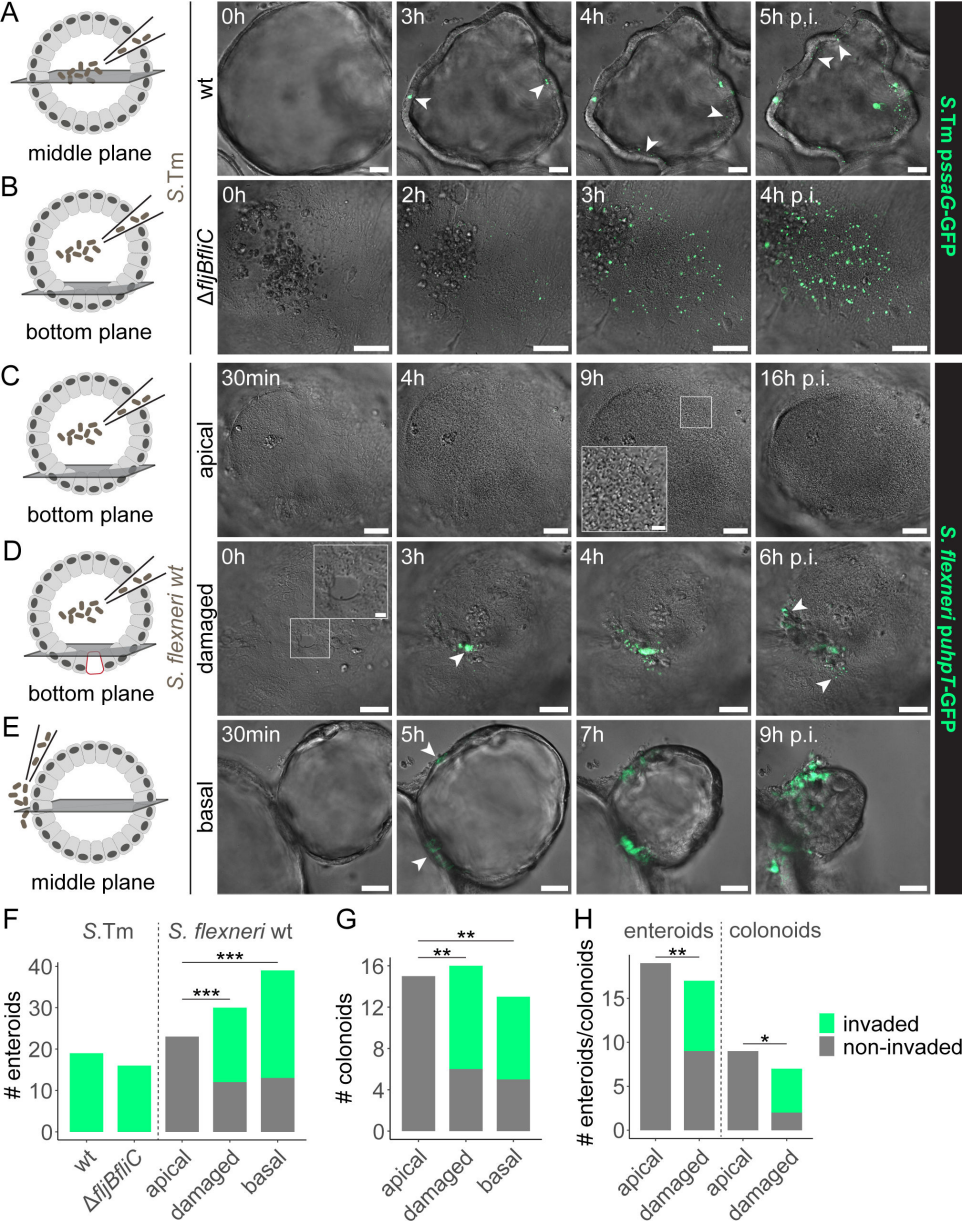
*Salmonella* efficiently colonizes the enteroid and colonoid epithelium from the apical side, but *Shigella* invasion requires epithelial damage. (**A and B**) Human enteroids were microinjected with (**A**) *Salmonella* wt and (**B**) Δ*fljBfliC* harboring the p*ssaG*-GFP intracellular reporter and imaged by wide-field differential interference contrast and fluorescence time-lapse microscopy. (**C–**E) Human enteroids/colonoids were infected with *Shigella* p*uhpT*-GFP via different routes and imaged as in panels A and B for up to 16 h p.i. (**C**) Upon luminal microinjection, *Shigella* accumulated at the bottom plane (see inset), but the unperturbed epithelium was not permissive for invasion from the apical side. (**D**) Epithelial damage introduced with the microinjection needle (see inset) resulted in localized invasion from the site of damage. Enteroids/colonoids invaded at the microinjection site were excluded from analysis. (**E**) Basal deposition of *Shigella* also resulted in successful epithelial colonization. (**F–H**) Quantification of the number of (**F**) enteroids, (**G**) colonoids, or (**H**) enteroids/colonoids upon enhanced absorptive IEC differentiation, invaded by either *Salmonella* strain and/or *Shigella* wt via the indicated routes of infection. Pooled counts from at least two independent experiments are shown. Statistical significance was determined by Pearson’s Chi-squared test with Bonferroni correction for multiple comparisons. In panel F, only enteroids infected with *Shigella* were included in the statistical analysis. *P < 0.05; **P < 0.01; and ***P < 0.001. Arrowheads indicate invasion foci. Scale bars: 50 µm (10 µm for insets). *S*. Tm, *Salmonella* Typhimurium.

Using this reporter approach, enteroid microinjections with motile *Salmonella* wild type (wt) revealed efficient IEC invasion at multiple sites all around the enteroids (Fig. 1A). Flagella-deficient *Salmonella* Δ*fljBfliC* and *Shigella* wt (naturally non-flagellated), on the other hand, accumulated atop the epithelium at the enteroid bottom plane within minutes post-infection (p.i.). Abundant *Salmonella* Δ*fljBfliC* invasion foci (GFP+) appeared at the enteroid bottom plane at 2–4 h p.i. (Fig. 1B). This confirms previous results showing that *Salmonella* flagellar motility may facilitate but is not strictly required for successful IEC invasion of microinjected enteroids (62). *Shigella* wt, however, remained completely non-invasive even after 16 h p.i. (Fig. 1C). Wondering if epithelial damage could promote *Shigella* invasion, we used the microinjection needle to introduce small scratches (~1–2 IECs in size) in the epithelium at the enteroid bottom plane prior to luminal microinjection. Strikingly, *Shigella* invaded the epithelium of these damaged enteroids as effectively as when the inoculum was deposited directly at the basal enteroid surface (positive control). This showed that epithelial damage enables *Shigella* colonization from the apical face of the enteroid epithelium by providing access to the basolateral IEC membrane (Fig. 1D through F). *Shigella* infection of colonoids from another donor via these different routes confirmed that the requirement for epithelial damage or basolateral access for *Shigella* IEC invasion is not segment- or donor-specific (Fig. 1G; Fig S2A through C). Furthermore, apical invasion at sites of epithelial damage also occurred when absorptive IEC differentiation of the enteroids and colonoids was enhanced (Fig. 1H; Fig S2D and E; see Materials and Methods for details), confirming that this phenotype does not depend on the extent of IEC differentiation.

### Non-flagellated, SiiE adhesin-deficient *Salmonella* phenocopies *Shigella* in its inability to stably adhere to the apical face of the enteroid and colonoid epithelium

**Fig 2:**
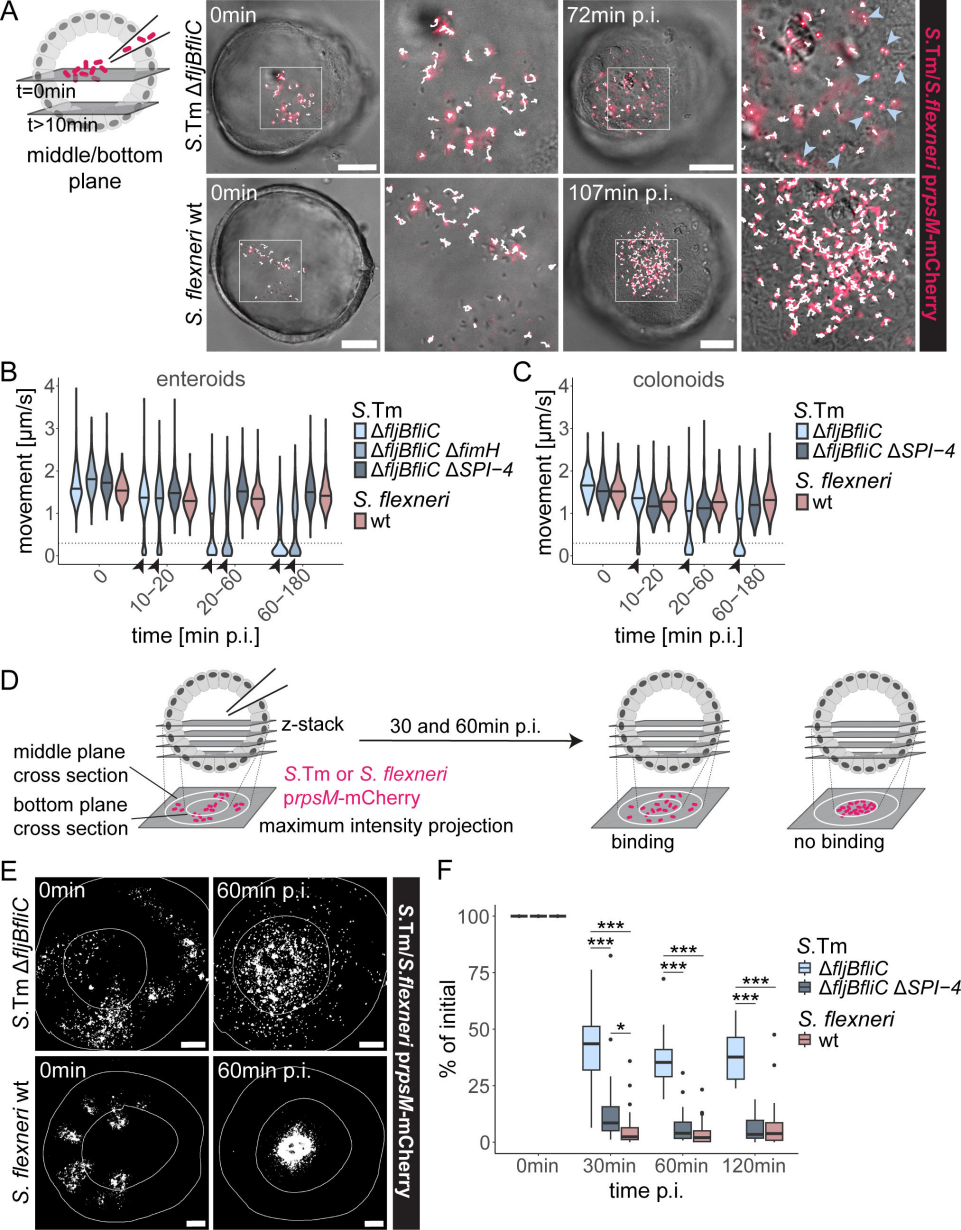
Non-flagellated SPI-4-deficient *Salmonella* and *Shigella* wt fail to stably attach to the apical face of the enteroid epithelium. (**A–C**) Enteroids were microinjected with the indicated *Salmonella* and *Shigella* strains harboring the constitutive p*rpsM*-mCherry reporter, and bacterial movement within the enteroids was tracked at the indicated time points p.i. (**A**) Schematic of the experimental setup and representative images of bacterial tracking (tracks in white). Stably adhering bacteria (indicated by light blue arrowheads) were observed for *Salmonella* Δ*fljBfliC* but not *Shigella* wt. Scale bars: 50 µm. (**B and C**) Quantification of *Salmonella* and *Shigella* movement speeds at different time points p.i. in microinjected (**B**) enteroids or (**C**) colonoids. The bimodal population distribution for *Salmonella* Δ*fljBfliC* and Δ*fljBfliC* Δ*fimH* with movement speeds close to 0 indicates a stably adhering subpopulation. Data from at least (**B**) 14 enteroids and 580 tracks per time point and strain, or (**C**) 9 colonoids and 430 tracks per time point and strain are shown. Horizontal lines indicate the median. Arrowheads indicate the presence of a non-moving subpopulation. (**D**) Schematic of the experimental setup: z-stacks of microinjected enteroids from the middle to the bottom plane were acquired at the indicated time points p.i. Middle and bottom plane cross-sections were outlined based on the DIC channel, and bacterial fluorescence (p*rpsM*-mCherry) retained at the side epithelium (outer ring), indicative of adhesion, was quantified as mCherry-positive area in maximum intensity projections. For a detailed description of the quantification method, refer to “Materials and Methods.” (**E**) In maximum intensity projections with middle and bottom plane outlines, *Salmonella* Δ*fljBfliC* but not *Shigella* wt fluorescence is retained at the side epithelium. Scale bars: 50 µm. (**F**) Quantification of fluorescence retained at the side epithelium relative to the initial mCherry-positive area at the sides. Data from at least 11 enteroids per strain are shown. In the box plots, the height of the boxes represents the interquartile range, whereas the horizontal line depicts the median. Whiskers extend to the most extreme data point within 1.5× IQR from the lower or upper boundary of the box. Outliers are indicated as dots. Statistical significance was determined by two-way ANOVA with Tukey’s HSD *post hoc* test. Significance for all comparisons per time point is indicated. *P < 0.05; ***P < 0.001. *S*. Tm, *Salmonella* Typhimurium.

Due to the striking difference in *Salmonella* Δ*fljBfliC* and *Shigella* wt invasion efficiency at the apical face of the epithelium, we next sought to pinpoint underlying determinants. As a first step toward successful IEC invasion, pathogens have to achieve stable cell surface adhesion. In a newly designed adhesion assay, we tracked Brownian-like motion of constitutively fluorescent (p*rpsM*-mCherry [68]), non-motile bacteria atop the epithelial surface at the bottom plane of microinjected enteroids and colonoids. We anticipated that bacterial Brownian motion would cease upon stable surface binding. While *Salmonella* Δ*fljBfliC* displayed two distinct adhering and non-adhering subpopulations already 10–20 min p.i., *Shigella* wt movement did not change over time, and no stably adhering subpopulation was observed even at 60–180 min p.i. (Fig. 2A through C; Movie S1). To assess if specific *Salmonella* adhesins were required for stable IEC binding, we mutated the prominent adhesins FimH (Δ*fljBfliC* Δ*fimH* [11]) or SiiE (Δ*fljBfliC* Δ*SPI-4* [14]) in the non-flagellated *Salmonella* strain. The absence of SPI-4/SiiE indeed completely abolished stable apical *Salmonella* adhesion (Fig. 2B and C; no obvious effect of FimH deletion, Fig. 2B). The SiiE dependence was observed in the jejunal enteroids and colonoids (Fig. 2B and C) and also in ileal enteroids established from yet another donor (Fig. S3A). Moreover, a T3SS-1-deficient *Salmonella* Δ*fljBfliC* Δ*invG* strain still managed to stably adhere, in contrast to the corresponding T3SS-deficient *Shigella* Δ*mxiD* strain (Table S1) (Fig. S3B). Finally, comparing non-motile *Salmonella* Δ*motA* Δ*SPI-4* (lacks the flagellar motor protein MotA, but maintains structural flagella [69]) with the non-flagellated *Salmonella* Δ*fljBfliC* Δ*SPI-4* ruled out adhesive properties of flagella in the absence of motility and SPI-4/SiiE (Fig. S3C). These data suggest that *Salmonella* Δ*fljBfliC* Δ*SPI-4* phenocopies *Shigella* wt in failing to stably adhere to the apical human IEC surface and emphasize the key role for SPI-4/SiiE in *Salmonella* adherence.

As an orthogonal approach to assess adhesion, we next quantified non-motile bacterial binding to the internal sides of non-centrally microinjected enteroids. Z-stacks of microinjected enteroids were acquired, and bacterial fluorescence retained at the enteroid sides was quantified over time (Fig. 2D). Our hypothesis was that only stable adhesion-competent bacteria would remain at this location, whereas non-binding bacteria should accumulate at the bottom. While ~40% of the initial *Salmonella* Δ*fljBfliC* or *Salmonella* Δ*fljBfliC* Δ*invG* fluorescence was retained at the enteroid sides, *Salmonella* Δ*fljBfliC* Δ*SPI-4, Shigella* wt, and *Shigella* Δ*mxiD* accumulated virtually exclusively at the bottom plane with ≤5% of the fluorescence retained at the sides (Fig. 2E and F; Fig. S3D; Movie S1). This fully corroborates our results from the adhesion assay above (Fig. 2A through C).

In the natural context of motile *Salmonella* wt, we wondered whether flagellar motility could generate enough momentum to gain access to the IEC membrane for T3SS-1-dependent stable docking, even in the absence of SiiE. To that end, parallel enteroid microinjections with motile *Salmonella* wt and *Salmonella ΔSPI-4* were executed. Bacterial movements were tracked over time to assign bacteria to three distinct subpopulations (motile: >5 µm/s, non-motile but exhibiting Brownian motion: 0.3–5 µm/s, and stably bound/invaded: <0.3 µm/s). Notably, *Salmonella ΔSPI-4* still displayed some extent of stable binding (Fig. S3E; 7% vs 46% for *Salmonella* wt at 60–180 min p.i.), while binding was abolished in the non-motile strain counterpart (Fig. S3C; 0.11% moving at <0.3 µm/s for *Salmonella* Δ*motA* Δ*SPI-4* at 60–180 min p.i.). Hence, the SiiE adhesin (strongly) and flagellar motility (weakly) contribute to making *Salmonella* a highly efficient apical IEC surface binder, in sharp contrast to *Shigella*.

Finally, we quantified bacterial invasion frequencies in 2D enteroid/colonoid-derived IEC monolayers that allow for the elimination of non-invaded bacteria by gentamicin and provide a stable imaging plane. We confirmed the use of the vacuolar p*ssaG*-GFP reporter to assess *Salmonella* invasion in the 2D monolayers (Fig. S4). In line with the results from both adhesion assays, *Salmonella* wt and *Salmonella* Δ*fljBfliC* invaded monolayers with relatively high efficiency, while invasion by *Salmonella ΔSPI-4* was reduced (Fig. S5A and B). Increased MOI (200) and a centrifugation step to force contact with the epithelial surface allowed for rare and unevenly distributed IEC invasion foci by *Salmonella* Δ*fljBfliC ΔSPI-4* and *Shigella* wt (Fig. S5C).

Altogether, these results indicate that *Salmonella* harbors an efficient “apical targeting module” that hinges on the SPI-4-encoded SiiE adhesin and flagellar motility and, combined with T3SS-1, enables IEC entry from the lumen at appreciable frequency. The absence of a corresponding apical targeting module in *Shigella* makes this bacterium reliant on cooperation between T3SS and external factors, such as preexisting epithelial damage (demonstrated here), or the presence of M cells (24, 25, 58, 70, 71), to establish an initial foothold in the epithelial lining.

### Efficient expansion of the intraepithelial *Shigella* population compensates for low adhesion and invasion frequencies relative to *Salmonella*

**Fig 3:**
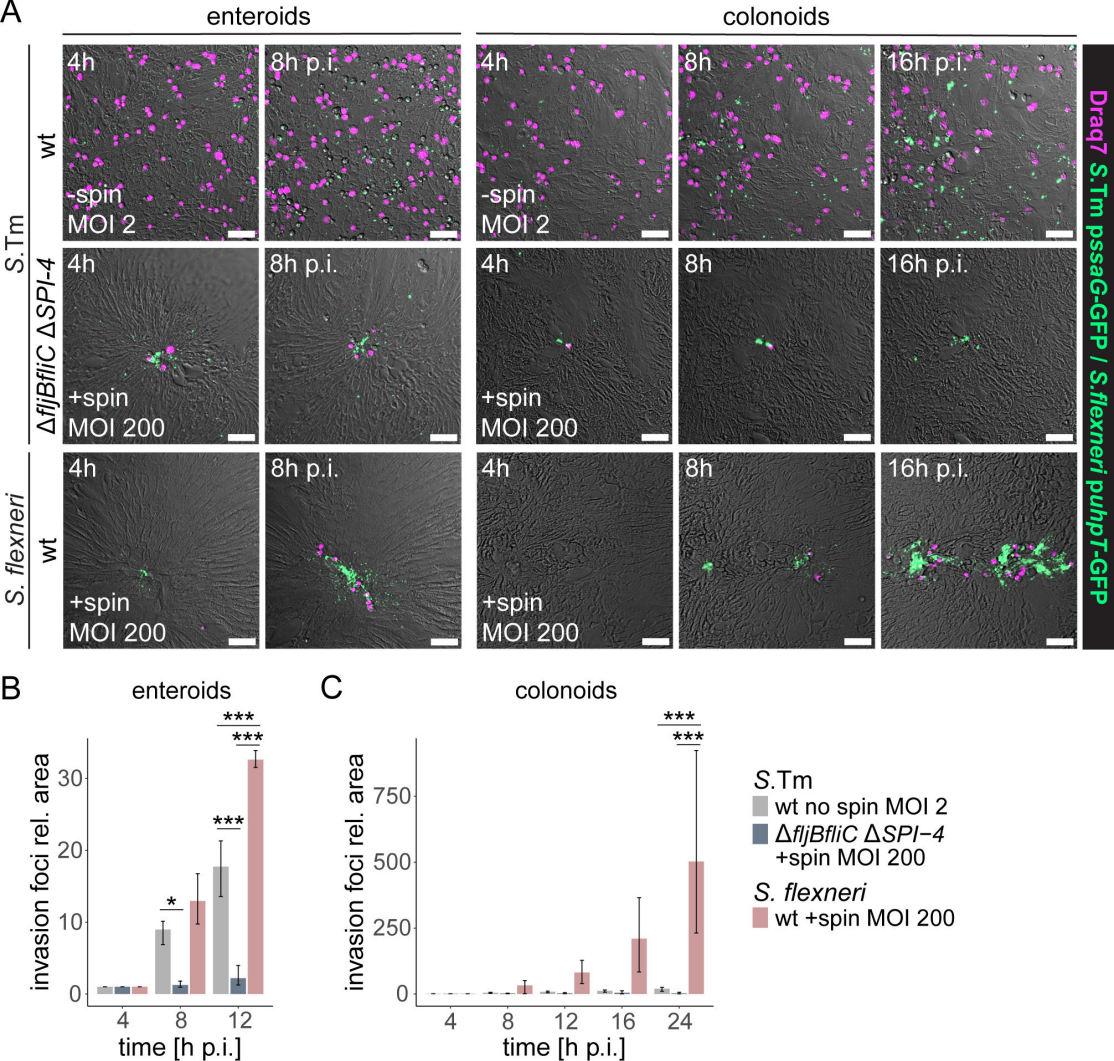
Efficient expansion of the intraepithelial *Shigella* population compensates for rare invasion events. (**A–C**) Enteroid- and colonoid-derived monolayers were infected with *Salmonella* wt (MOI 2), *Salmonella* Δ*fljBfliC* Δ*SPI-4,* and *Shigella* wt (MOI 200 + centrifugation), harboring the intracellular p*ssaG*-GFP or p*uhpT*-GFP reporters, respectively, in the presence of the membrane-impermeable nuclear dye Draq7. Individual invasion foci were followed by time-lapse microscopy, and their expansion was quantified. (**A**) Representative images of abundant invasion foci and Draq7-positive cells observed for *Salmonella* wt, rare and confined invasion foci by Δ*fljBfliC* Δ*SPI-4,* and intraepithelial expansion of rare foci with limited Draq7 signal for *Shigella* wt. Scale bars: 50 µm. (**B and C**) Quantification of the GFP-positive area relative to the 4 h p.i. time point reveals efficient intraepithelial expansion for *Shigella* in both (**B**) enteroid- and (**C**) colonoid-derived monolayers. Data are plotted as mean + range of three replicates per strain, with one replicate corresponding to the mean of 6–10 fields of view for an individually infected well. Statistical significance was determined by two-way ANOVA with Tukey’s HSD *post hoc* test. Significance for comparisons among all strains at the respective time point is indicated. *P < 0.05; ***P < 0.001.

We noted that the vastly higher numbers of IEC invasion events of apical targeting module-proficient *Salmonella* were associated with induction of high levels of IEC death, as revealed by holes in infected monolayers (Fig. S5C; in line with our previous observations [56, 62]). Therefore, the fate of intraepithelial *Salmonella* and *Shigella* after IEC invasion was further investigated. We conducted time-lapse imaging of individual invasion foci in enteroid/colonoid-derived IEC monolayers and included Draq7 as a marker for IEC death and permeabilization (72). As revealed by the abundant Draq7 signal, *Salmonella* wt infection caused prompt induction of IEC death (Fig. 3A), which often started even before maturation of the intracellular p*ssaG*-GFP bacterial reporter. By combining a high MOI and centrifugation, occasional IEC invasion events could be identified and traced for *Shigella* wt and the corresponding “apical targeting module-deficient” *Salmonella* Δ*fljBfliC ΔSPI-4* strain. Notably, while these two strains again phenocopied each other during the early infection stages, *Shigella* wt expanded over time (12–24 h p.i.) with dramatically higher efficiency within both enteroid and colonoid epithelia (Fig. 3A through C). *Shigella* wt induced only low levels of IEC death during this intraepithelial expansion phase (Fig. 3A). Prompt cell death induction by *Salmonella* wt and rare *Shigella* wt invasion events, followed by efficient intraepithelial spread, were also confirmed in differentiation-enhanced enteroid/colonoid-derived monolayer cultures (Fig. S6). Hence, *Shigella* (but not *Salmonella* Δ*fljBfliC ΔSPI-4*) compensates for the extremely low adhesion and invasion capacity by efficient post-invasion expansion within the epithelium and minimal induction of IEC death.

### The concerted action of OspC3 and IcsA enables *Shigella* to outrun the epithelial inflammasome cell death response by lateral spread

**Fig 4:**
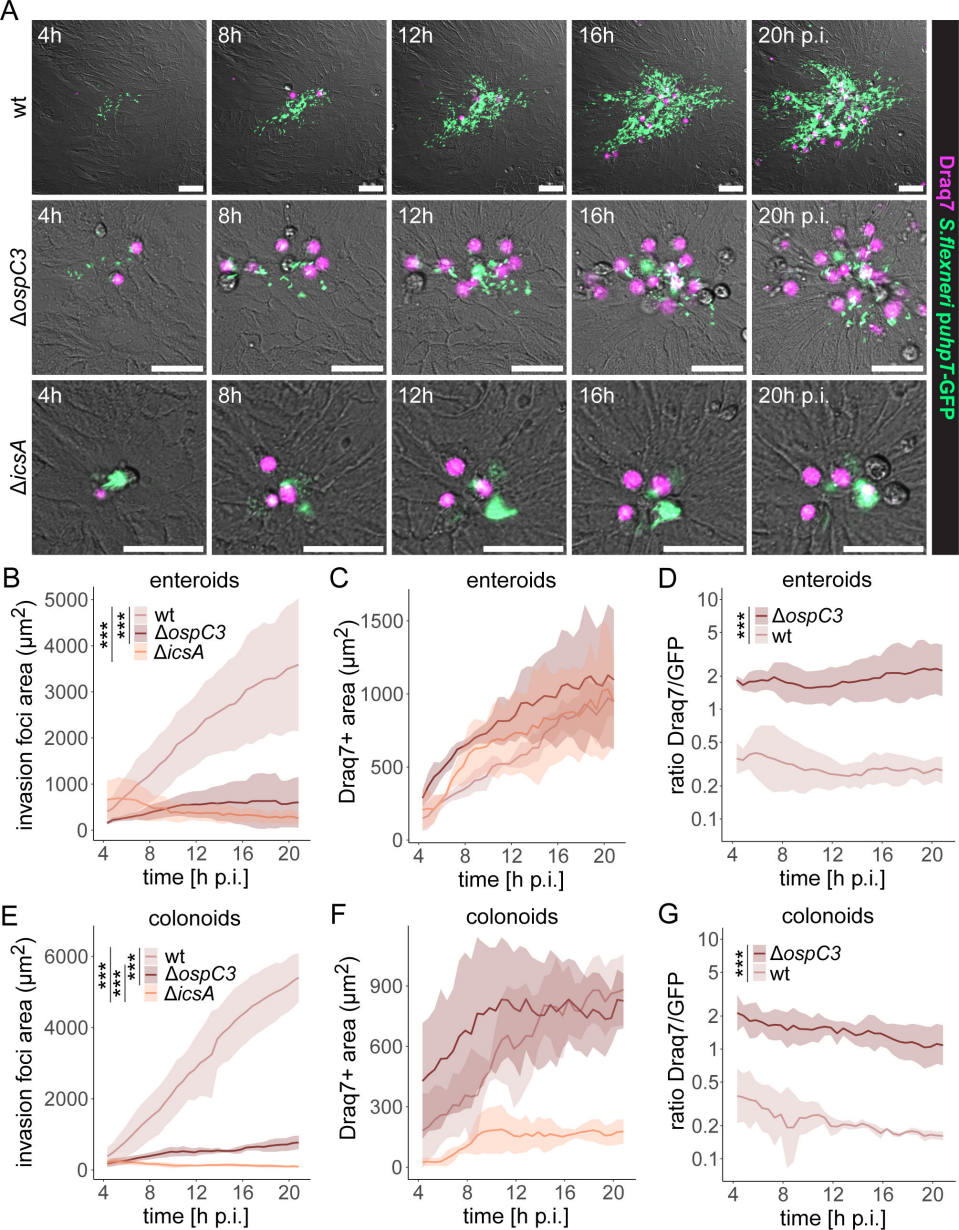
*Shigella* OspC3 suppresses IEC death long enough to enable IcsA-dependent intraepithelial spread. (**A–D**) Enteroid-derived monolayers were infected with the indicated *Shigella* strains harboring the p*uhpT*-GFP reporter (MOI 200 + centrifugation) in the presence of Draq7. Individual invasion foci were followed by time-lapse microscopy, and their expansion was quantified. (**A**) Representative images of *Shigella* wt, but not *Shigella* Δ*ospC3* or *Shigella* Δ*icsA*, expanding intraepithelially. Scale bars: 50 µm. Quantification of (**B**) the GFP-positive area and (**C**) Draq7-positive area over time. (**D**) The increased Draq7-to-GFP ratio for *Shigella* Δ*ospC3* indicates that a reduced IEC death frequency couples to successful intraepithelial expansion. Data are plotted as mean + standard deviation (SD) of three replicates per strain, with one replicate corresponding to the mean of 3–11 foci in an individually infected well. (**E–G**) Colonoid-derived monolayers were infected and imaged as in panels A–D, and (**E**) GFP-positive area, (**F**) Draq7-positive area, and (**G**) Draq7-to-GFP ratio were quantified over time. Data are plotted as mean + SD of three replicates per strain, with one replicate corresponding to the mean of 2–8 foci in an individually infected well. Statistical significance was determined by (**B and E**) two-way ANOVA with Tukey’s HSD *post hoc* test, or (**D and G**) two-way ANOVA, with significance for the factor “strain” indicated. ***P < 0.001.

Based on the radically different fates of intraepithelial *Salmonella* and *Shigella* populations, we next addressed the interplay between *Shigella* intraepithelial expansion and induction of IEC death and pinpointed the virulence factors involved. Within this effort, 2D enteroid/colonoid-derived monolayers were infected with *Shigella* wt, *Shigella* Δ*ospC3* lacking a T3SS effector previously reported to suppress non-canonical (Caspase-4/11) inflammasome activation (OspC3 [35, 36, 54]), or *Shigella* Δ*icsA* deficient for the nucleator for cytosolic actin polymerization and cell-to-cell spread (IcsA [32–34]). While *Shigella* wt spread efficiently within the enteroid epithelium, *Shigella* Δ*ospC3* and *Shigella* Δ*icsA* did not. Such invasion foci were instead frequently cleared from the epithelium by the induction of cell death (Fig. 4A and B; Movie S3). The total Draq7-positive area was broadly comparable for *Shigella* wt and *Shigella* Δ*ospC3* infections (Fig. 4C). However, as invasion foci differed dramatically in size (Fig. 4B), the Draq7-to-GFP ratio was analyzed as a measure for the probability of cell death induction per infected IEC. This ratio was markedly higher for *Shigella* Δ*ospC3* than for *Shigella* wt over the entire imaging period (Fig. 4D; 4–20 h p.i.). Essentially identical results were obtained in colonoid-derived monolayers (Fig. 4E through G). In addition, we tested the role of two other *Shigella* T3SS effectors, IpaH7.8 and IpaH9.8, shown to inhibit inflammasome activation downstream (40) or upstream (37–39, 55) of Caspase-4/11, respectively, finding that neither effector was strictly required for intraepithelial expansion (Fig. S7). This shows that OspC3-dependent delay of human IEC death allows sufficient time for IcsA-dependent lateral *Shigella* spread and thereby successful intraepithelial expansion.

The Caspase-4-dependent non-canonical inflammasome is largely responsible for cell death induction in enterobacterium-infected human intestinal epithelia, but the NAIP/NLRC4 inflammasome (not targeted by OspC3) plays the most prominent role in mouse IECs (47–51, 53). Consequently, WT and *Nlrc4^-/-^* murine enteroid-derived monolayers were infected with *Shigella* wt, incubated with Draq7, and followed by time-lapse microscopy. This revealed the essential absence of stable intraepithelial *Shigella* foci paired with prompt induction of IEC death in WT monolayers, in sharp contrast to lower levels of cell death and efficient *Shigella* foci expansion in *Nlrc4^-/-^* monolayers (Fig. S8). The results highlight that a failure of inflammasome-dependent cell death permits rapid lateral *Shigella* expansion also in murine epithelia.

**Fig 5:**
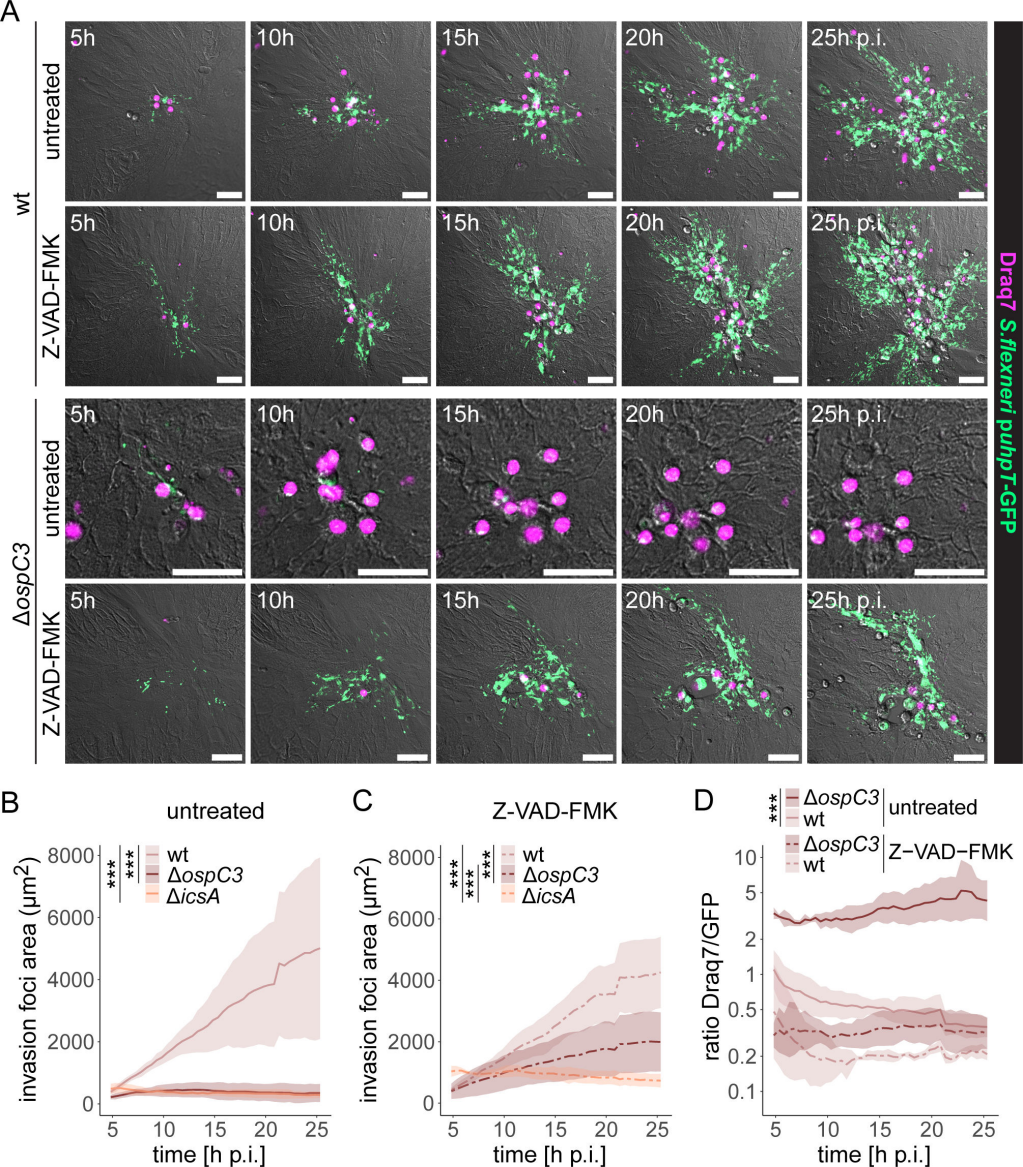
The failure of a *Shigella* Δ*ospC3* mutant to expand intraepithelially can be restored by chemical caspase inhibition. (**A–D**) Enteroid-derived monolayers were infected with the indicated *Shigella* p*uhpT*-GFP strains as in Fig. 4 in the presence or absence of the broad-spectrum caspase inhibitor Z-VAD-FMK, and individual invasion foci were followed by time-lapse microscopy. (**A**) Representative images of Z-VAD-FMK treatment restoring *Shigella* Δ*ospC3* intraepithelial expansion to wt levels. Scale bars: 50 µm. (**B and C**) Quantification of the GFP-positive area in (**B**) untreated and (**C**) Z-VAD-FMK-treated monolayers over time. (**D**) The Draq7-to-GFP ratio for *Shigella* Δ*ospC3* was reduced to wt levels upon Z-VAD-FMK treatment. Data are plotted as mean + SD of three replicates per strain and treatment, with one replicate corresponding to the mean of 3–10 (untreated) or 5–12 (Z-VAD-FMK) foci in an individually infected well. Statistical significance was determined by two-way ANOVA with Tukey’s HSD *post hoc* test. Significance for comparisons among all strains for the respective treatment is indicated. ***P < 0.001.

Finally, when human enteroid/colonoid-derived monolayers were treated with the broad-spectrum caspase inhibitor Z-VAD-FMK, *Shigella* Δ*ospC3* intraepithelial expansion and a correspondingly low Draq7-to-GFP ratio were restored, while the treatment did not affect *Shigella* wt or *Shigella* Δ*icsA* colonization (Fig. 5, enteroids; Fig. S9, colonoids). This formally demonstrates that inflammasome-dependent, caspase-mediated cell death restricts *Shigella* cell-to-cell spread. However, when cell death is delayed to a certain threshold, either chemically or by a T3SS effector, the intraepithelial *Shigella* population accomplishes lateral expansion. Altogether, these data highlight that *Shigella* compensates for its poor apical IEC invasion capacity by the evolution of an efficient “intraepithelial expansion module” that combines cell death delay by OspC3 and IcsA-mediated cell-to-cell spread to escape the IEC counter response.

## DISCUSSION

Previous work on enterobacterial virulence factors and epithelial responses has provided a rich catalog of infection-relevant biochemical interactions. These studies have placed strong emphasis on molecular mechanisms. However, less attention has been paid to recapitulating primary cell behavior and resolving temporal interconnections between virulence factor sets and host cell responses throughout infection stages. Time-lapse imaging in human enteroids and colonoids that retain primary IEC features and functional cell death responses has allowed us to generate a dynamic, spatiotemporally resolved map of the divergent *Salmonella* and *Shigella* epithelial colonization strategies.

**Fig 6:**
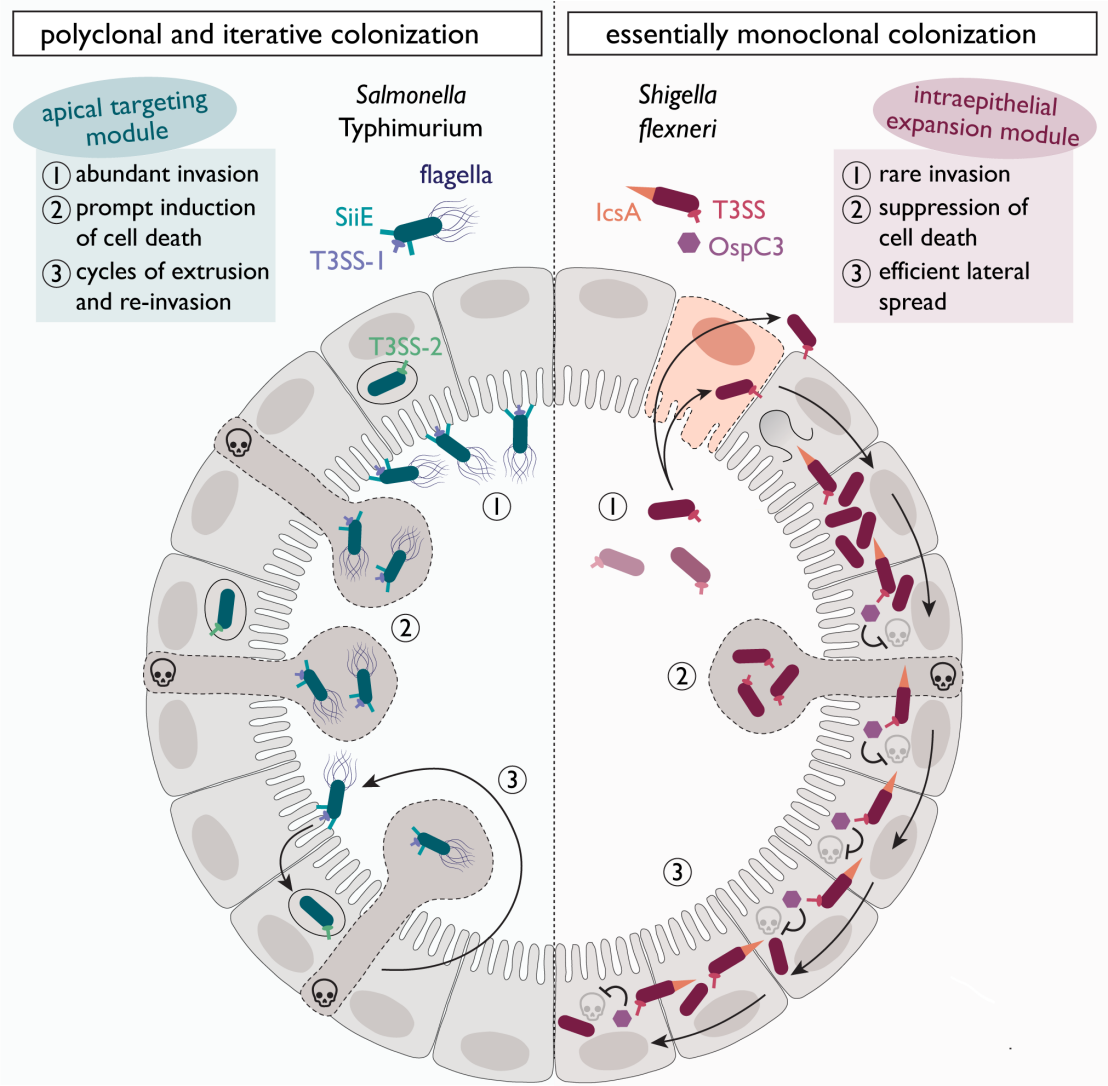
A conceptual model highlighting the basis for divergent *Salmonella* and *Shigella* colonization strategies of human intestinal epithelia. In the *Salmonella* “polyclonal and iterative” colonization strategy (left side), flagellar motility and the SPI-4-encoded adhesin system cooperate with T3SS-1 to form a highly efficient apical targeting module. This results in productive apical epithelial surface scanning and binding and abundant invasion foci. However, invasion events are typically abrogated by the prompt induction of IEC death, which results in fast and iterative cycles of IEC invasion and extrusion. The *Shigella* “essentially monoclonal” colonization strategy (right side), on the other hand, is dependent on an intraepithelial expansion module that comprises the inflammasome-inhibitory T3SS effector OspC3 and the actin nucleator IcsA. The concerted action of these effectors allows *Shigella* to laterally outrun the consequences of the IEC death response and compensate for exceptionally rare invasion events by efficient intraepithelial expansion. The red cell with dashed outlines indicates a damaged IEC that allows for *Shigella* access to the basolateral membrane.

Equipped with an apical targeting module that combines flagellar motility and adhesin activity, *Salmonella* efficiently scans the epithelial surface and encounters a high density of suitable binding sites. The SPI4-encoded SiiE adhesin system, which we identified as central for *Salmonella* attachment to the apical enteroid/colonoid surface, has previously been attributed an adhesive role for invasion of polarized cell lines and *in vivo* in mice (14, 29). Here, we extend these findings by using uniquely designed adhesion assays to reveal how SiiE enables transition from Brownian-like floating atop the epithelial surface to stable adhesion, likely accomplished through multivalent binding to glycosylated membrane-bound mucins like MUC1 and MUC13 (73, 74). This step preceded and enabled T3SS-1-driven invasion. *Salmonella* expresses a range of other adhesins under various conditions (10–13), so the prominent role of SiiE identified here could be replaced by other adhesins in different host species. Notably, the importance of SPI-4/SiiE-dependent binding appears consistent across our experiments in human jejunal and ileal enteroids, as well as colonoids. While flagella have also been ascribed host cell membrane-adhesive properties (8, 9), our assays provided no evidence for such function in apical binding to non-transformed human IECs. Rather, flagellar motility allowed *Salmonella* to partially overcome the adhesin requirement, likely by generating momentum to penetrate the apical brush border and stably dock to the IEC membrane via T3SS-1. Collectively, the collaboration of three virulence systems, flagella, a SPI-4-encoded adhesin, and T3SS-1, mediates *Salmonella* targeting, stable docking, and apical IEC invasion at a reasonable frequency in human enteroids/colonoids (Fig. 6).

Removal of the apical targeting module (*ΔfljBfliC ΔSPI-4*) effectively transformed *Salmonella* into a *Shigella*-like particle, stalled floating atop the IEC surface. This highlights the lack of well-described adhesins and flagella as the decisive differences between *Shigella* and *Salmonella* during early IEC invasion. Moreover, it suggests that cooperation with external factors is needed for *Shigella* to establish an initial foothold in human absorptive epithelia. Indeed, this notion is supported by other *in vitro* models for *Shigella* infection that rely on artificial adhesin expression or a centrifugation step to enforce host cell contact and appreciable numbers of invasion events (75, 76). The requirement for preexisting epithelial damage to colonize the enteroid/colonoid epithelium from the apical side in our microinjection model, supposedly by exposing the basolateral membrane for T3SS docking, offers one mechanism of how this barrier could be breached *in vivo*. In addition, transit through M cells has been described as an important first entry portal for *Shigella* both *in vivo* and in enteroid/colonoid models (24, 25, 28, 58, 71). Other studies have also shown that infiltrating polymorphonuclear leukocytes (28, 64), malnourishment (59), EGTA treatment (22, 23), or extruding IECs (77) may allow for bacterial access into the intraepithelial niche. Furthermore, host factors have been suggested to directly (20, 27) or indirectly (17–19) promote *Shigella* adhesion. Nevertheless, *Shigella* apical invasion of non-transformed human gut absorptive epithelium by own efforts appears exceedingly rare, which highlights the IEC binding/invasion step as a major barrier for *Shigella* infection (Fig. 6).

At the post-invasion step, we found that the concerted action of the inflammasome antagonist OspC3 and the actin nucleator IcsA uniquely allowed for lateral *Shigella* spread just in time to evade IEC death. In the absence of OspC3, *Shigella* invasion foci were frequently cleared, but caspase inhibition by chemical means again allowed the pathogen to overcome the epithelial cell death response and maintain an infection focus. While the non-canonical Caspase-4 pathway targeted by OspC3 represents the main inflammasome response in human IECs (47, 49, 53), the dominant NAIP/NLRC4-Caspase-1 pathway in the murine epithelium (47, 48, 50) is not shut off by *Shigella* effectors. This dichotomy in epithelial inflammasomes prevents *Shigella* colonization of the murine gut epithelium, as shown in a recent study (46), and temporally explained here by our live-cell imaging experiments in murine enteroid-derived monolayers.

Collectively, we describe two virulence factor modules that give rise to divergent strategies for successful colonization of human absorptive intestinal epithelia by prominent representatives of invasive enterobacteria. The *Salmonella* “polyclonal and iterative” colonization strategy relies on the apical targeting module and is vastly superior in all steps prior to entry. This results in multiple independent but short-lived IEC invasion foci. Inflammasome-driven IEC extrusion fuels luminal reseeding and expansion of bacteria primed for repeating rounds of reinvasion (62, 78, 79), thereby achieving prolonged epithelial colonization (Fig. 6). *Shigella*, on the other hand, utilizes an “essentially monoclonal” colonization strategy marked by rare IEC invasion events paired with superiority in steps subsequent to entry. Here, the identified intraepithelial expansion module constituted by OspC3 and IcsA pairs temporal delay of the epithelial inflammasome response with exploitation of the host cell actin machinery to achieve cell-to-cell spread just in time before a colonized IEC succumbs (Fig. 6). It is, moreover, noteworthy that the sensitive cell death responses of primary IECs do not permit the expansive intracellular replication seen in cell lines, but the *Shigella* strategy still enables sufficient rounds of intra-IEC replication for plaque expansion. Finally, considering host range, it is tempting to speculate that host-restricted epithelial invaders such as *Shigella* are more likely to adapt a recognition-suppressive strategy subsequent to IEC entry, whereas generalist invaders such as *Salmonella* would favor high efficiency at the entry step. This is due to the infeasibility of suppressing immune recognition pathways that can differ markedly across host species.

Despite primary IEC features and cell death responses in enteroids/colonoids, the only partial IEC maturation state, the absence of fully established soluble mucus, and the lack of microbiota and immune cells should be pointed out among the limitations of this study. The absence of extraepithelial factors might also explain the lack of segment specificity for infection in enteroid/colonoid models, as previously pointed out by others (23, 24). Of note, enterobacterial pathogens oftentimes target sites devoid of a continuous mucus layer, such as in the murine cecum (6), or the follicle-associated epithelium featuring a penetrable mucus layer (80). More advanced organoid models that better incorporate one or several of the abovementioned aspects are continuously being developed (64, 81–85) and will be valuable for future studies building on this one. The current setup nevertheless allowed us to follow *Salmonella* and *Shigella* epithelial colonization in non-transformed human epithelia in real time. This revealed how modules of bacterial species-specific virulence factors active at the apical targeting step (*Salmonella*) vs the intracellular expansion step (*Shigella*) dictate vastly different epithelial colonization dynamics (Fig. 6).

## MATERIALS AND METHODS

### Bacterial strains, plasmids, and culture conditions

All bacterial strains used in this study are listed in Table S1. *Salmonella enterica* serovar Typhimurium (*Salmonella*, *S*. Tm) strains were of SL1344 background (SB300, streptomycin resistant, Sm^R^) (86). Besides the wild type, previously described Δ*invG*, Δ*SPI-4,* and Δ*fljBfliC* mutants were used. The Δ*fljBfliC* Δ*fimH,* Δ*fljBfliC* Δ*SPI-4,* Δ*fljBfliC* Δ*invG,* and Δ*motA* Δ*SPI-4* mutants were generated by flip out of the kanamycin resistance cassette (Kan^R^) from the aforementioned Δ*fljBfliC* and previously published Δ*motA* strains, followed by transfer of previously described deletions from *Salmonella* 14028 (C0831, Δ*fimH*; C1793, Δ*invG*) (87) or SL1344 strains (Δ*SPI-4*) (14), respectively, by P22 transduction. Genotypes were verified by PCR using primers k1 and k2 along with the respective strain-specific screening primers (Table S2). All *Shigella flexneri* (*Shigella*, *S. fl*) strains were of M90T background (88). Besides the wt, the previously described Δ*mxiD* mutant was used. Δ*ospC3,* Δ*icsA*, Δ*ipaH7.8,* and Δ*ipaH9.8* mutants were generated by lambda red recombination (89), transforming M90T pSIM5 (90) with the PCR product obtained using plasmid pKD13 as template and the respective deletion primers (Table S2). Genotypes were verified by PCR using primers k1 and k2 along with the respective strain-specific screening primers (Table S2). Reporter plasmids, namely the constitutive pFPV-mCherry (p*rpsM*-mCherry; Addgene plasmid number 20956) (68), intracellular vacuolar reporter pM975 (p*ssaG*-GFPmut2) (50, 66), and intracellular cytosolic reporter p*uhpT*-GFP are listed in Table S1. Reporter plasmid p*uhpT*-GFP has similarity to p*uhpT*-mCherry as described and validated earlier (67) and comprises a pBR322 origin of replication, an ampicillin resistance cassette (Amp^R^), and the GFP-mut2 open reading frame downstream of the *uhpT* promoter. For infections, *Salmonella* strains were grown overnight at 37°C for 12 h in LB/0.3 M NaCl (Sigma-Aldrich) with appropriate antibiotics and sub-cultured (1:20 dilution) in the same medium without antibiotics at 37°C for 4 h. *Shigella* strains were grown overnight at 30°C for 16 h in LB with appropriate antibiotics and sub-cultured (1:50 dilution) in LB without antibiotics at 37°C for 2 h until OD_600_ ~ 0.7. For Caco-2 infections, the inoculum was reconstituted in DMEM GlutaMAX (Gibco)/0.1 mM non-essential amino acids (Gibco) at a concentration of 2 × 10^7^ CFU/mL, while for enteroid/colonoid infections, it was reconstituted in antibiotic-free human (hOGM/hODM) or mouse (mOGM) IntestiCult organoid growth/differentiation medium (StemCell) at a concentration of ~10^9^ CFU/mL and further diluted 1:100 for low MOIs where indicated.

### Human enteroid and colonoid establishment

Human jejunal enteroids and colonoids were established as previously described (62, 91). Newly established enteroids and colonoids were frozen at passages 1–4 by gently dissolving Matrigel (Corning) domes in ice-cold DMEM/F12 (Gibco)/0.25% BSA (Gibco) and resuspending the enteroids/colonoids in DMEM/F12/10% heat-inactivated fetal bovine serum (FBS) (Gibco)/10% DMSO (Sigma-Aldrich), followed by freezing in a Mr. Frosty Freezing container at −80°C overnight and transfer to liquid nitrogen gas phase for cryopreservation.

### Human and murine enteroid/colonoid culture

Human enteroids/colonoids were cultured in Matrigel (75%) domes overlaid with hOGM/1× PenStrep (Gibco) at 37°C and 5% CO_2_ and passaged once a week as previously described (72). Enteroids from passages 4 to 30, and colonoids from passages 3 to 16 were used for experimentation. WT (92) and *Nlrc4^-/-^* (52, 93) murine jejunal enteroids of C57BL/6 background were cultured in Matrigel (60%) domes overlaid with mOGM/1× PenStrep at 37°C and 5% CO_2_ and passaged every 5–7 days as previously described (72), and passages 10–16 were used for experimentation. Where indicated, enteroids/colonoids were cultured in hODM (StemCell) without DAPT for 4–5 days to enhance absorptive IEC differentiation.

### Caco-2 cell culture and infection

Caco-2 cells were cultured in DMEM/10% heat-inactivated FBS/0.1 mM NEAA/1× PenStrep at 37°C and 10% CO_2_ and passaged every 2–3 days. One day prior to infection, cells were seeded at ~55,000 cells/cm^2^ in a glass-bottom 24-well plate (Cellvis) pre-coated with 100 µg/cm^2^ collagen I (Corning). For infection, the medium was exchanged for half the volume of the well of antibiotic-free DMEM/0.1 mM NEAA, and wells were filled by adding the same volume of the prepared inoculum. The plate was centrifuged at 700 *g* for 10 min, incubated at 37°C and 5% CO_2_ for 40 min, and washed three times with DMEM/10% FBS/0.1 mM NEAA before the addition of DMEM/10% FBS/0.1 mM NEAA/200 µg/mL gentamicin (Sigma-Aldrich).

### Human enteroid/colonoid microinjection

Microinjection of *Salmonella* and *Shigella* into human enteroids and colonoids was performed as previously described (62). Briefly, enteroids/colonoids were passaged, embedded in Matrigel (~100%) in 35 mm glass-bottom dishes (MatTek), and overlaid with 2 mL antibiotic-free hOGM. The culture medium was replaced every 3 days and prior to microinjection. Enteroids/colonoids were injected 5–8 days after passaging. Microinjection needles were prepared from 1.0 mm filamented glass capillaries (World Precision Instruments; no. BF100-78-10; Borosilicate, 1 mm wide, 100 mm long, with filament) using a micropipette puller (Sutter Instruments; P-1000) and the following settings: heat = ramp + 5; pull = 60; velocity = 80; delay = 110; and pressure = 200. Needles were beveled at a 30° angle on a fine-grit diamond lapping wheel, loaded with the *Salmonella* or *Shigella* inocula by fluidic force and mounted on a microinjector (MINJ-FLY; Tritech Research) controlled by a micromanipulator (uMP-4; Senapex). Pressured air pulses (20–200 ms) were applied to inject ~50–500 bacteria, depending on the size of the enteroid/colonoid.

### Establishment and infection of 2D human enteroid/colonoid-derived monolayers

Human enteroid and colonoid-derived monolayers were established on Matrigel-coated polymer coverslips (µ-Slide 8-well high Cat. No 80806 or µ-Plate 96-well black Cat. No 89626, Ibidi) as previously described (72). Enteroids/colonoids 7 days after passaging were extracted from Matrigel domes using Gentle Cell Dissociation Reagent (StemCell), washed once in DMEM/F12/1.5% BSA, and dissociated into single cells using TrypLE (Gibco) and mechanical shearing by pipetting. The single cell suspension was washed once in DMEM/F12/1.5% BSA, reconstituted in antibiotic-free hOGM/10 µM Y-27632 (Sigma-Aldrich), and seeded out at 750,000 cells/cm^2^ in wells pre-coated with a 1:40 Matrigel dilution in DPBS (Gibco). Monolayers were maintained at 37°C and 5% CO_2_, and 2–3 days post-establishment, the medium was exchanged for fresh antibiotic-free hOGM without Y-27632. Where indicated, monolayers were cultured in hODM for 2 days prior to infection to enhance absorptive IEC differentiation. Monolayers were used for infection 3–5 days post-establishment (6–7 days post-establishment for hODM-grown monolayers). After washing with DMEM/F12, half the volume of the well of antibiotic-free hOGM/hODM, supplemented with 100 µM Z-VAD-FMK (AH diagnostics) where indicated, was added, and the wells were filled by adding the same volume of the prepared inoculum. Unless indicated otherwise, slides were centrifuged at 700 *g* for 10 min prior to incubation at 37°C and 5% CO_2_ for 40 min to allow for bacterial invasion. After washing three times with DMEM/F12/400 µg/mL gentamicin, hOGM/hODM, supplemented with 50 µg/mL gentamicin, 0.75 µM Draq7 (Invitrogen), and/or 50 µM Z-VAD-FMK where indicated, was added to the wells.

### Establishment and infection of 2D murine enteroid-derived monolayers

For monolayer establishment, murine enteroids were pre-treated with mOGM/1 mM valproic acid (Cayman chemicals)/3 µM CHIR99021 (Cayman chemicals) (CV medium) for 7 days as previously described (72, 91). Enteroids were extracted from Matrigel domes using Gentle Cell Dissociation Reagent, washed once in DMEM/F12/1.5% BSA, and dissociated into single cells using TrypLE and mechanical shearing by pipetting. The single cell suspension was washed once in DMEM/F12/1.5% BSA, reconstituted in antibiotic-free CV medium/10 µM Y-27632, and seeded out at 530,000 cells/cm^2^ in wells pre-coated with a 1:40 Matrigel dilution in DPBS. Monolayers were maintained at 37°C and 5% CO_2_, and 24–36 h post-establishment, the medium was exchanged for fresh antibiotic-free mOGM without Y-27632. Monolayers were used for infection 3 days post-establishment. After washing with DMEM/F12, half the volume of the well of antibiotic-free mOGM was added, and the wells were filled by adding the same volume of the prepared inoculum. Slides were centrifuged at 700 *g* for 10 min, incubated at 37°C and 5% CO_2_ for 40 min, and washed three times with DMEM/F12/400 µg/mL gentamicin, followed by the addition of mOGM/50 µg/mL gentamicin/0.75 µM Draq7.

### Fixation and staining of enteroid/colonoid-derived monolayers

At 3–6 h p.i., enteroid and colonoid-derived monolayers were washed twice with DPBS and fixed with DPBS/2% paraformaldehyde for 15 min in the dark. Following two additional washes with DPBS, they were stained with 1 µg/mL DAPI (Sigma-Aldrich) and 1 U/mL Phalloidin-Alexa647 or 1 U/mL Phalloidin-Alexa488 (Molecular Probes) in DPBS/0.1% Triton X-100 (Sigma-Aldrich) for 30 min, shaking in the dark. After two final washes as described above, DPBS was added to the wells prior to image acquisition.

### Microscopy

Imaging was performed on a custom-built microscope based on an Eclipse Ti2 body (Nikon), using 60×/0.7 and 40×/0.6 Plan Apo Lambda air objectives (Nikon) and a back-lit sCMOS camera with pixel size 11 µm (Prime 95B; Photometrics). For time-lapse microscopy, the imaging chamber was maintained at 37°C and 5% CO_2_ in a moisturized atmosphere. Bright-field images were acquired using differential interference contrast (DIC), while fluorescence images were obtained using the excitation light engine Spectra-X (Lumencor) and emission collected through a quadruple band pass filter (89402; Chroma). Infected Caco-2 cells were imaged every 2 min for 16 h. Microinjected enteroids and colonoids were imaged immediately after microinjection, and time-lapse images were acquired every 5–15 min for up to 16 h. For bacterial tracking, microinjected enteroids and colonoids were imaged at 500-ms intervals for 20 frames in total at each indicated time point. Z-stacks of non-centrally injected enteroids and colonoids were acquired between the middle and bottom planes at the indicated time points with 2–5 µm between slices. Manually detected invasion foci in enteroid/colonoid-derived monolayers were imaged every 30 min for up to 25 h. Automated image acquisition of fixed enteroid-derived monolayers was performed using Micromanager 2.0 (94), while fixed colonoid-derived monolayers were imaged manually to include rare and unevenly distributed invasion foci.

### Image analysis

Background subtraction for fluorescence channels was performed in Fiji (a version of ImageJ) (95), applying a rolling ball radius of 50 pixels. The TrackMate plugin (96) in Fiji was used for tracking, and all tracks were verified manually due to bacterial crowding at the bottom plane, complicating automated tracking. For quantification of the fluorescence retained at the side epithelium of microinjected enteroids, maximum intensity projections of background-subtracted z-stacks from the mCherry channel were generated in Fiji, and middle and bottom plane cross-sections were delineated based on DIC images. The same threshold was set for all time points from one replicate, and the fluorescence above threshold for the non-overlapping area of the middle and bottom plane cross-sections (i.e., the side epithelium) was quantified in Fiji. A CellProfiler (97) customized pipeline was used for the quantification of invasion foci in fixed enteroid/colonoid-derived monolayers. Holes in the monolayers observed under some conditions were not subtracted from the total area of the FOV. For the quantification of invasion foci and Draq7-positive areas, one threshold value per channel was used for all time-lapse movies from one experiment, and the area above threshold was quantified in Fiji.

### Statistical analysis

All data were analyzed and plotted using the tidyverse collection of R packages (98) in RStudio (99). Where applicable, statistical significance was determined by Pearson’s Chi-squared test using the functions chisq.test() and pairwise_chisq_gof_test() (rstatix package; [100]) or two-way ANOVA with Tukey’s honestly significant difference *post hoc* test using the functions aov() and TukeyHSD() in RStudio.

## SUPPLEMENTAL MATERIAL

Supplemental material mbio.00911-25-s0001.pdf Supplemental figures and tables; legends for supplemental movies.

Movie S1 mbio.00911-25-s0002.avi Salmonella Δ*fljBfliC* but not *Shigella* wt stably adheres to the epithelium at the enteroid bottom plane.

Movie S2 mbio.00911-25-s0003.avi *Salmonella* Δ*fljBfliC* but not *Shigella* wt stably adheres to the side epithelium of microinjected enteroids.

Movie S3 mbio.00911-25-s0004.avi *Shigella* wt but not Δ*ospC3* or Δ*icsA* expands and spreads efficiently within enteroid-derived monolayers.

ASM does not own the copyrights to Supplemental Material that may be linked to, or accessed through, an article. The authors have granted ASM a non-exclusive, world-wide license to publish the Supplemental Material files. Please contact the corresponding author directly for reuse.
