# Supplementary material for: Determinants of divergent Salmonella and Shigella epithelial colonization strategies resolved in human enteroids and colonoids
Source: mBio. 2025 May 30;16(7):e00911-25. doi: 10.1128/mbio.00911-25 (PMC12239579; doi:10.1128/mbio.00911-25)
Supplement: Supplemental material — Supplemental figures and tables; legends for supplemental movies. [file mbio.00911-25-s0001.pdf]

Supplementary material for:

Determinants of Divergent *Salmonella* and *Shigella* Epithelial Colonization Strategies Resolved in Human Enteroids and Colonoids

Petra Geiser[a], Maria Letizia Di Martino[a], Ana C. C. Lopes[a], Alexandra Bergholtz[a], Magnus Sundbom[b], Martin Skogar[b], Wilhelm Graf[b], Kajsa Björner[c], Johan Vessby[c], Dominic-Luc Webb[c,d], Per M. Hellström[c], Jens Eriksson[a] and Mikael E. Sellin[a,e]#

[a]*Department of Medical Biochemistry and Microbiology, Uppsala University, Uppsala, Sweden*
[b]*Department of Surgical Sciences, Uppsala University, Uppsala, Sweden*
[c]*Department of Medical Sciences, Uppsala University, Uppsala, Sweden*
[d]*DLW Bioanalytics, Märsta, Sweden*
[e]*Science for Life Laboratory, Uppsala, Sweden*
#Correspondence: mikael.sellin@imbim.uu.se

SUPPLEMENTARY FIGURES

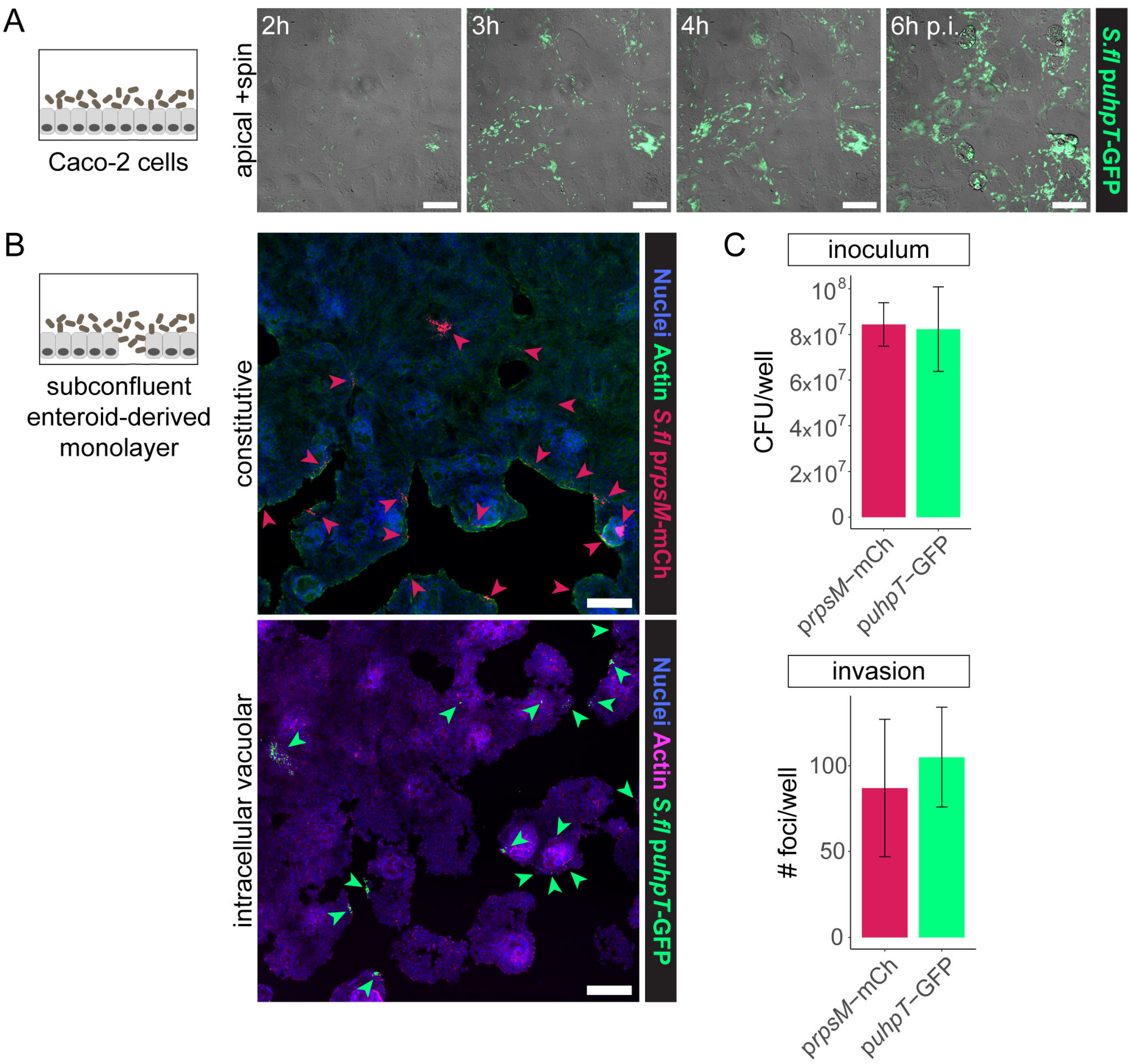

Supplementary Figure S1. Verification of the intracellular cytosolic p*uhpT*-GFP reporter for *Shigella*. (A) Caco-2 cells were apically infected with *Shigella* wt p*uhpT*-GFP (MOI 140) for 40 min, including a centrifugation step, and imaged by DIC and fluorescence time-lapse microscopy. (B-C) Subconfluent human enteroid-derived monolayers were apically infected with *Shigella* wt harboring either the constitutive p*rpsM*-mCherry or the intracellular cytosolic p*uhpT*-GFP at MOI 200 for 40 min including a centrifugation step. Monolayers were extensively washed and fixed at 6 h p.i. and stained with DAPI (nuclei) and Phalloidin (actin). (B) Arrow heads indicate invasion foci. Scale bars: 200 µm. (C) The quantification of the inoculum size (CFU/well, top) and number of invasion foci/well (bottom) reveals no difference between the constitutive and intracellular cytosolic reporter (statistical significance assessed by Mann-Whitney U test, p=0.8857), suggesting that the vast majority of intracellular *Shigella* expresses the p*uhpT*-GFP reporter and that this reporter accurately quantifies the intraepithelial *Shigella* population. Data is plotted as mean + SD of 4 replicates. *S.fl*, *Shigella flexneri*.

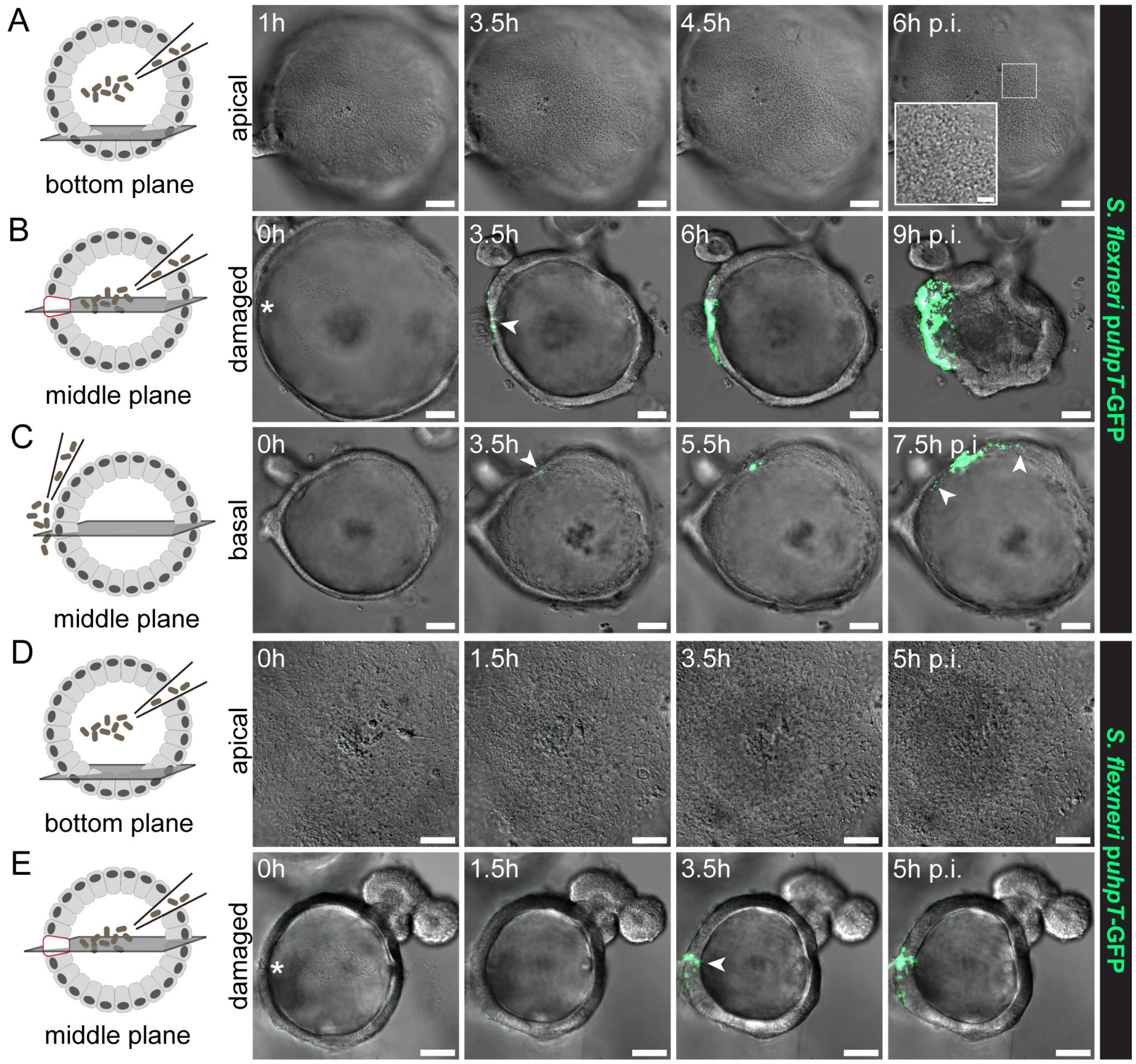

Supplementary Figure S2. Epithelial damage results in localized *Shigella* invasion of the colonoid epithelium from the apical side. (A-C) Human colonoids were infected with *Shigella* wt p*uhpT*-GFP via different routes and imaged by DIC and fluorescence time-lapse microscopy. (A) Upon luminal microinjection, *Shigella* quickly accumulates at the bottom plane (see insert), but the unperturbed epithelium was not permissive for invasion from the apical side. (B) Epithelial damage introduced with the microinjection needle (*) resulted in localized invasion from the site of damage. (C) Basal deposition of *Shigella* also resulted in successful epithelial colonization. (D-E) Human colonoids upon enhanced absorptive IEC differentiation were microinjected with *S. flexneri* p*uhpT*-GFP in the presence or absence of epithelial damage (*) as in A-B. (D) While *S. flexneri* was unable to invade the unperturbed apical colonoid surface, (E) epithelial damage (*) promoted epithelial access for invasion. Arrowheads indicate invasion foci. Scale bars: 50µm (10µm for insert).

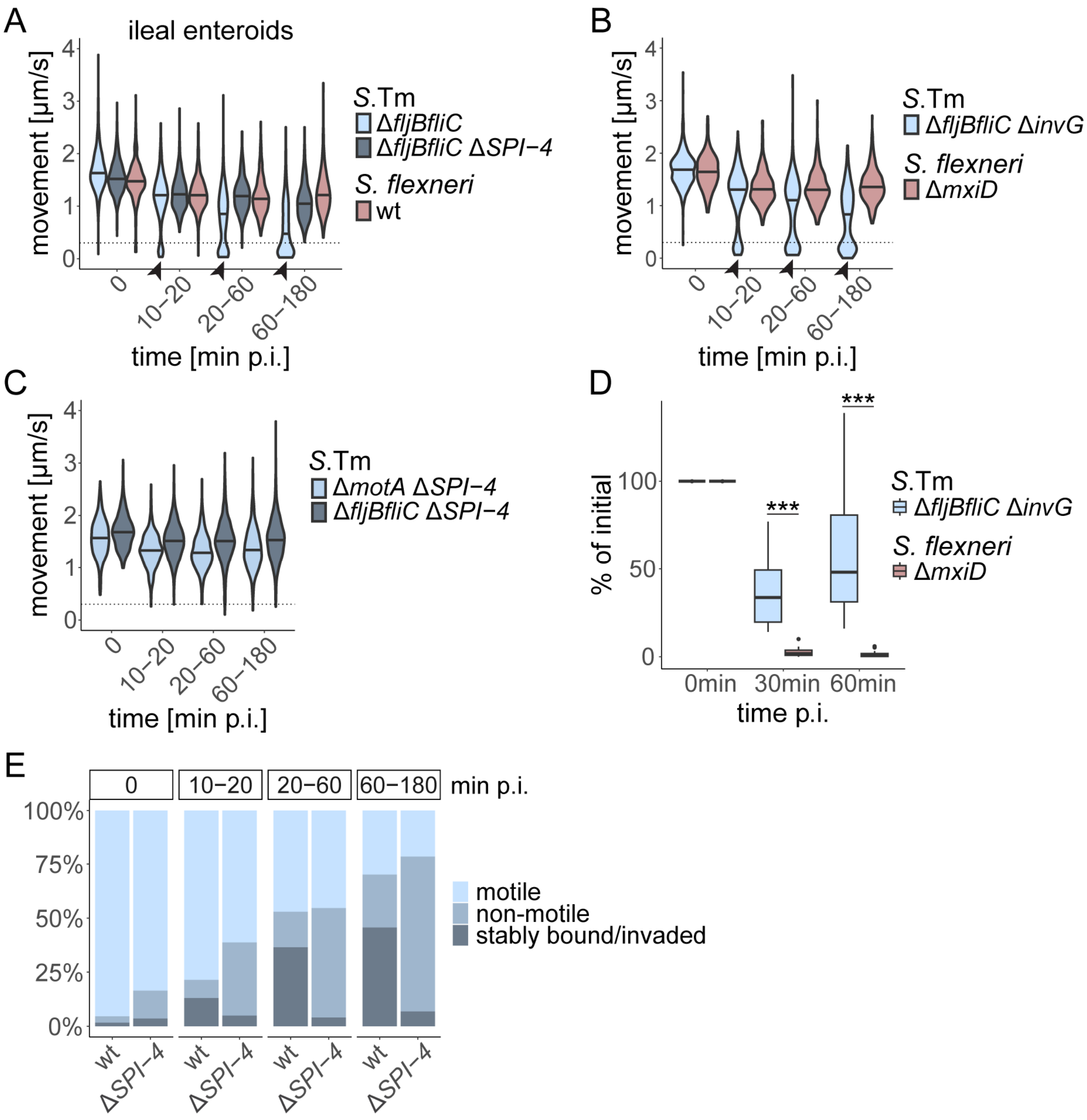

Supplementary Figure S3. **The SiiE adhesin and flagellar motility promote adhesion to the enteroid and colonoid epithelium.** (A-D) Enteroids were microinjected with the indicated *Salmonella* and/or *Shigella* strains harboring the constitutive p*rpsM*-mCherry reporter, and bacterial movement speeds at the indicated time points p.i. were quantified. (A) Microinjection in ileal enteroids reveals a stably adhering subpopulation at time points >0 min p.i. for adhesin-proficient *Salmonella* Δ*fljBfliC*, but not adhesin-deficient *Salmonella* Δ*fljBfliC* Δ*SPI-4* or *Shigella* wt. (B) The bimodal population distribution for *Salmonella* Δ*fljBfliC* Δ*invG* with movement speeds close to 0 indicates a stably adhering population in the absence of a structural T3SS-1, (C) while no stably adhering subpopulation was observed in the absence of the SPI-4-encoded adhesin SiiE, regardless of the presence (Δ*motA*) or absence (Δ*fljBfliC*) of structural flagella. Data from at least (A) 7 enteroids and 460 tracks per time point, (B) 8 enteroids and 230 tracks per time point and strain, (C) or 15 enteroids and 490 tracks per time point and strain. Horizontal lines indicate the median. Arrowheads indicate the presence of a non-moving subpopulation. (D) Quantification of adhesion of T3SS-1-deficient *Salmonella* and *Shigella* strains to the side epithelium of non-centrally microinjected enteroids based bacterial p*rpsM*-mCherry fluorescence retained at the side epithelium as described in Figure 2D-F. *Salmonella* Δ*fljBfliC* Δ*invG*, but not *Shigella* Δ*mxiD* fluorescence, is retained at the side epithelium. Data from at least 16 enteroids

per strain is shown. In the box plots, the height of the boxes represents the interquartile range (IQR), whereas the horizontal line depicts the median. Whiskers extend to the most extreme data point within 1.5x the IQR from the lower or upper boundary of the box. Outliers are indicated as dots. Statistical significance was determined by two-way ANOVA with Tukey's HSD post hoc test. ***, P<0.001. *S*.Tm, *Salmonella* Typhimurium. (E) Enteroids were microinjected with *Salmonella* wt and *Salmonella* Δ*SPI-4* p*rpsM*-mCherry and bacterial movement within the enteroids was tracked. Tracks were classified as motile (>5µm/s), non-motile (0.3-5µm/s) or stably bound/invaded (<0.3µm/s) based on their swimming speeds and plotted as percentage of the total population. Data from at least 7 enteroids and at least 310 tracks per time point and strain.

<u>Interpretation of Figure S3A</u>: For ileal enteroid microinjections, some degree of bacterial aggregation, starting at 20-180 min p.i., was observed in 12-45% of the enteroids for all bacterial strains. The reason for this aggregation phenotype might be increased mucus or antimicrobial peptide production in ileal enteroids compared to jejunal enteroids or colonoids, and characterization of this phenomenon will be an interesting subject for further investigation. As bacterial aggregation prohibited bacterial tracking, the subfraction of ileal enteroids displaying bacterial aggregation were excluded from analysis. Beyond this phenomenon, the conclusions from these ileal enteroid microinjection experiments were fully in line with the results from jejunal enteroids and colonoids for all strains tested.

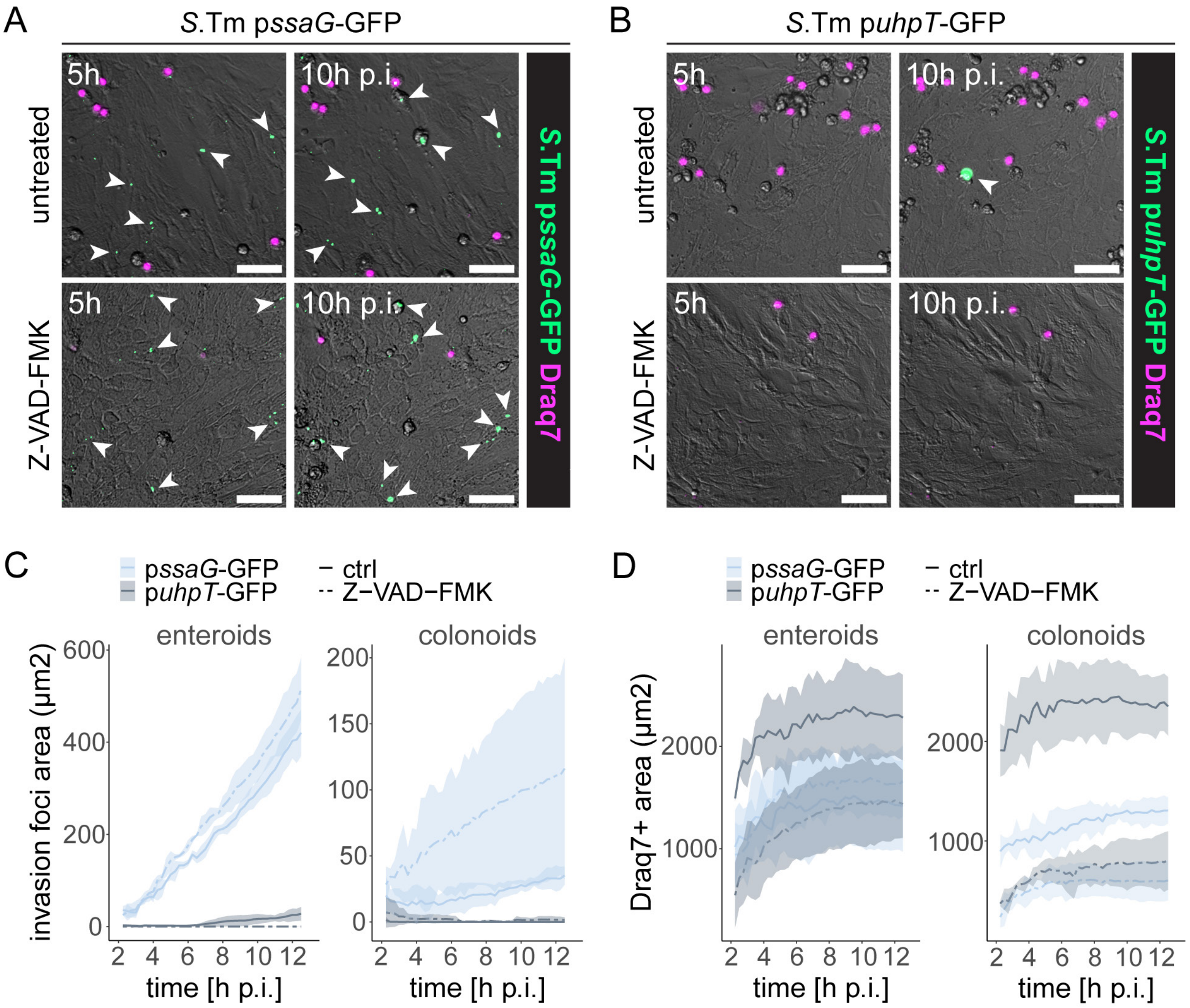

Supplementary Figure S4. Vacuolar and cytosolic fluorescent reporters to assess intraepithelial *Salmonella* populations in enteroid/colonoid-derived monolayers. Enteroid/colonoid-derived monolayers were infected with *Salmonella* wt harboring the vacuolar p*ssaG*-GFP, or the cytosolic p*uhpT*-GFP, reporter at MOI 2 for 20 min in the presence or absence of the broad-spectrum caspase inhibitor Z-VAD-FMK and IEC death was assessed by staining with the membrane-impermeable nuclear dye Draq7. (A) Abundant invasion foci could be observed with the vacuolar p*ssaG*-GFP reporter, (B) whereas p*uhpT*-GFP-positive cytosolic *Salmonella* were rarely observed regardless of treatment, which indicates that the cytosolic subpopulation is either very limited or too short lived to allow for fluorescent reporter maturation. Of note, most Draq7-positive, dying IECs do not harbor any GFP-positive bacteria for either reporter, suggesting that both reporters underestimate the intraepithelial *Salmonella* population. Arrow heads indicate invasion foci. Scale bars: 50µm. (C) Quantification of the invasion foci area confirms that the vacuolar p*ssaG*-GFP reporter most robustly detects intraepithelial *Salmonella*. (D) Quantification of the Draq7-positive area indicates that Z-VAD-FMK reduces, but not fully blocks, *Salmonella*-induced cell death in enteroid/colonoid-derived monolayers. (C-D) Data is plotted as mean + SD for 3 replicates per strain and condition, with one replicate corresponding to the mean of 4-7 fields of view for an individually infected well.

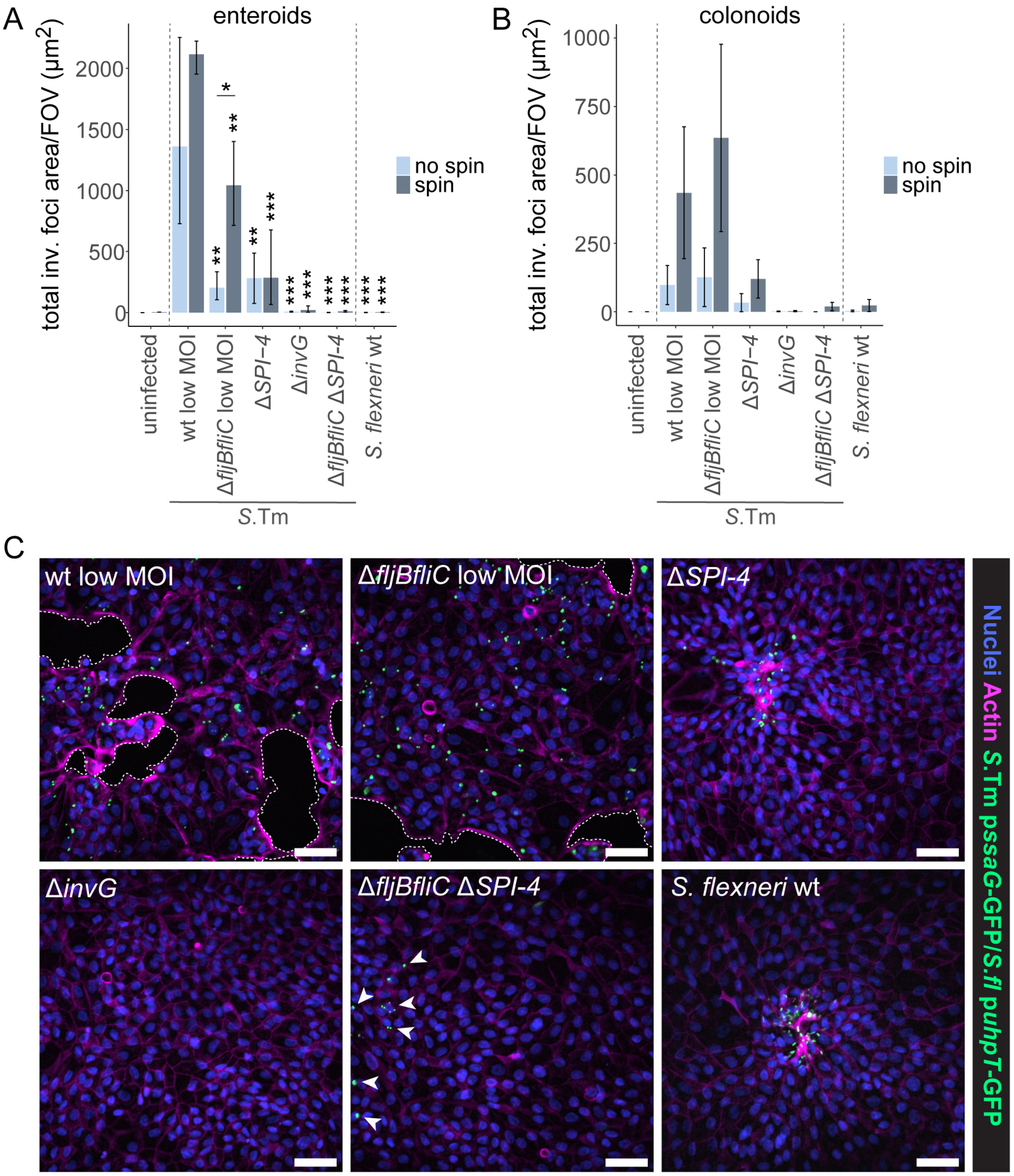

Supplementary Figure S5. **The SiiE adhesin and flagellar motility both promote invasion of the enteroid and colonoid epithelium.** (A) Enteroid-derived monolayers were infected with the indicated *Salmonella* and *Shigella* strains at MOI 200, or MOI 2-4 ("low MOI"), respectively, for 40min, with or without centrifugation. Monolayers were fixed at 3-4 h p.i. and the total area of invasion foci at randomly selected fields of view (FOVs) was determined based on the intracellular reporters p*ssaG*-GFP and p*uhpT*-GFP, respectively. Data is plotted as mean + range of 3 replicates per strain (1 replicate for uninfected control), with one replicate corresponding to the mean of 14-16 FOVs for an individually infected well. Statistical significance was determined by two-way ANOVA with Tukey's HSD post hoc test. Significance for comparison to *Salmonella* wt low MOI, and between 'no spin' and 'spin' condition for each strain is indicated. *, P<0.05; **, P<0.01; ***, P<0.001. (B-C) Colonoid-derived monolayers were infected as in A, but FOVs for analysis were selected manually to also include rare and unevenly

distributed invasion foci. (B) Data is plotted as mean + range of 2 replicates per strain (1 replicate for uninfected control without spin), with one replicate corresponding to the mean of 3-15 (no spin) or 6-17 (spin) FOVs for an individually infected well. Statistical significance was not assessed, as 2 replicates are insufficient. (C) Representative images of infected colonoid-derived monolayers (+ centrifugation) stained with DAPI (nuclei) and phalloidin (actin). Holes in the monolayers are outlined with dashed lines. Scale bars: 50µm. *S*.Tm, *Salmonella* Typhimurium; *S.fl*, *Shigella flexneri*.

Interpretation of Figure S5. Enteroid-derived monolayers were infected with different *Salmonella* and *Shigella* strains with or without a centrifugation step to force contact of the bacteria with the epithelial surface in order to determine the requirement for flagellar motility and the SPI-4 adhesin system for invasion under both conditions. The infections were allowed to proceed for 40 min such that even the non-motile bacteria could reach the surface of the monolayers by gravity in the absence of centrifugation. Adhesin-proficient *Salmonella* wt and *Salmonella* Δ*fljBfliC* invaded monolayers with high efficiency that further increased upon centrifugation, particularly for the non-flagellated strain (Fig S5A). Due to the observed reduction in bacterial binding, infections with adhesin-deficient *Salmonella* strains and *Shigella* were performed at 50-100x higher MOI. While invasion by *Salmonella ΔSPI-4* was lower than for the wt even at increased MOI, it was completely undetectable for *Salmonella* Δ*fljBfliC ΔSPI-4* and *Shigella* wt regardless of centrifugation (Fig S5A). This confirms previous results and highlights the importance of the *Salmonella* SPI-4 adhesin system not only for successful adhesion to, but also subsequent invasion of the enteroid epithelium, and that this requirement for adhesins can be partially circumvented by flagellar motility. When repeating the experiment in colonoid-derived monolayers and manually choosing the FOVs for analysis, it was observed that, upon centrifugation, even *Salmonella* Δ*fljBfliC ΔSPI-4* and *Shigella* wt were occasionally able to invade the epithelium, but these invasion foci were exceedingly rare and unevenly distributed with hotspots for invasion determined by monolayer topology (Fig S5B-C). Furthermore, it was found that although the invasion efficiency for *Salmonella ΔSPI-4* was higher than for its non-flagellated counterpart, invasion foci displayed a similarly uneven distribution (Fig S5C). Adhesion-competent *Salmonella* wt and *Salmonella* Δ*fljBfliC*, on the other hand, colonized the colonoid epithelium more evenly and efficiently, thereby also inducing significant levels of IEC death, as revealed by holes in the monolayers (Fig S5C).

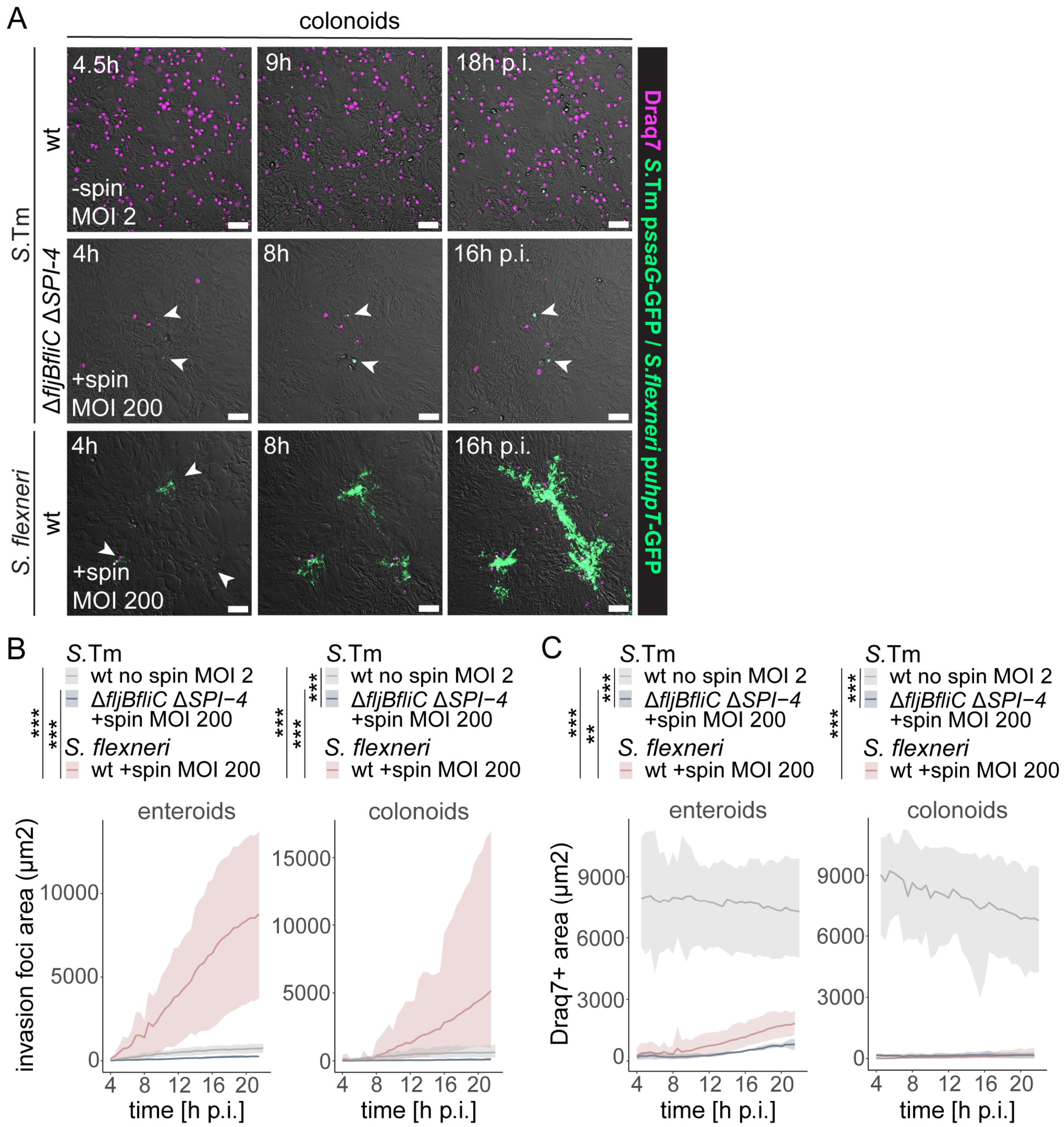

Supplementary Figure S6. The intraepithelial *Shigella* population efficiently expands within differentiation-enhanced enteroid/colonoid-derived monolayers to compensates for rare invasion events. Enteroid/colonoid-derived monolayers cultured in hODM to enhance absorptive IEC differentiation were infected with *Salmonella* wt (MOI 2), *Salmonella* Δ*fljBfliC* Δ*SPI-4* and *Shigella* wt (MOI 200 + centrifugation), harboring the intracellular p*ssaG*-GFP or p*uhpT*-GFP reporters, respectively, in the presence of Draq7. Individual invasion foci were followed by time-lapse microscopy and their expansion was quantified. (A) Representative images of abundant invasion foci and Draq7-positive cells observed for *Salmonella* wt, rare and confined invasion foci by *ΔfljBfliC ΔSPI-4*, and intraepithelial expansion of rare foci with limited Draq7 signal for *Shigella* wt. Arrow heads indicate invasion foci. Scale bars: 50µm. (B) Quantification of the GFP-positive area suggests efficient intraepithelial expansion for *Shigella*. (C) Quantification of the Draq7-positive area reveals prompt and abundant induction of IEC death upon *Salmonella* wt infection, and more limited cell death induction upon infection with *Salmonella ΔfljBfliC ΔSPI-4* or *Shigella* wt. (B-C) Data is plotted as mean + range of 3 replicates wells per strain with at least 3 fields of view per well (enteroids) or as mean + range of at

least 6 fields of view per strain acquired from at least 2 independently infected wells (colonoids). Statistical significance was determined by two-way ANOVA with Tukey's HSD post hoc test. **, P<0.01; ***, P<0.001.

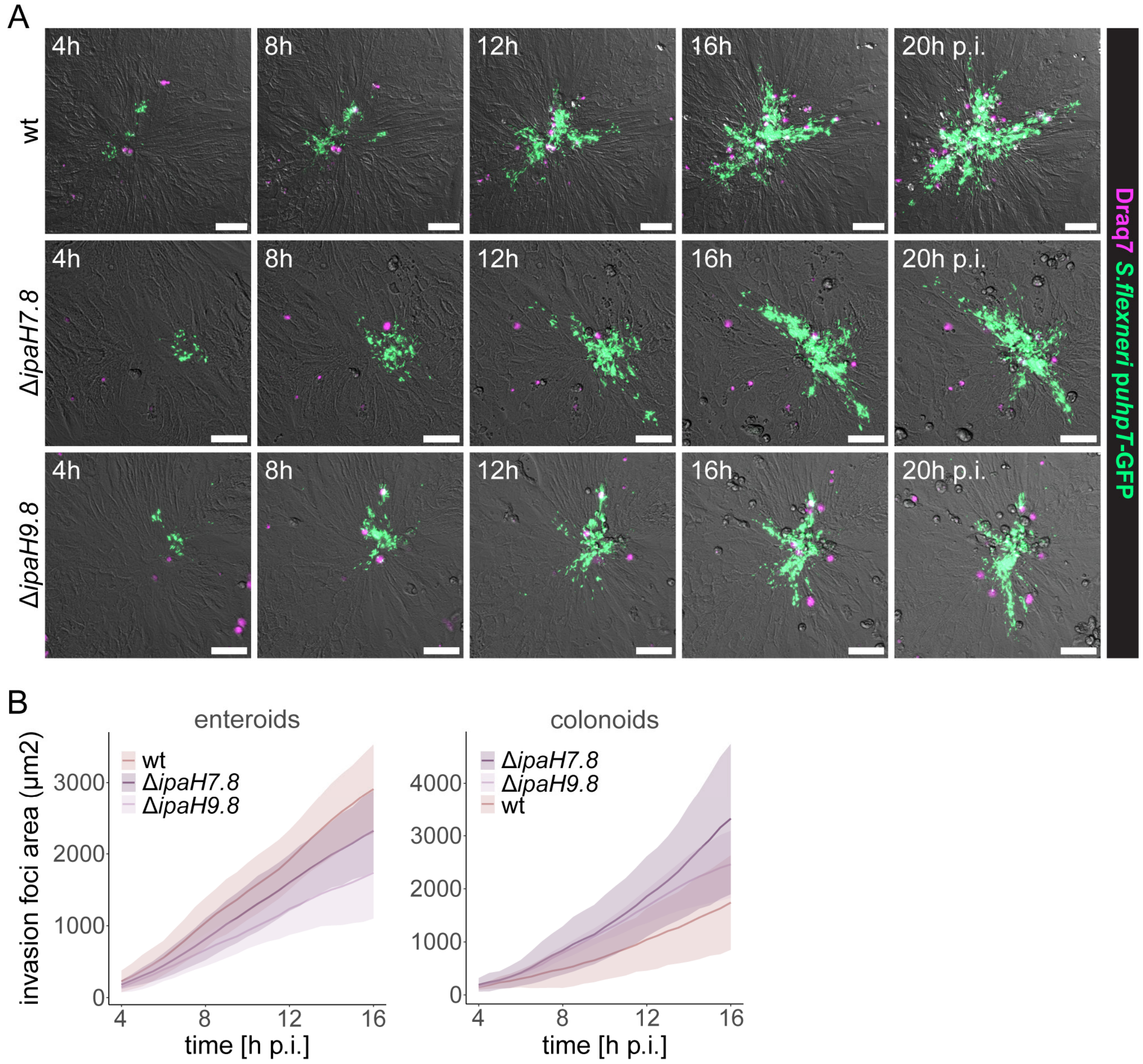

Supplementary Figure S7. ***Shigella*** **IpaH7.8 and IpaH9.8 are not required for successful intraepithelial expansion.** Enteroid/colonoid-derived monolayers were infected with the indicated *Shigella* strains harboring the p*uhpT*-GFP reporter (MOI 200 + centrifugation) and stained with Draq7. Individual invasion foci were followed by time-lapse microscopy and their expansion was quantified. (A) Representative images of *Shigella* wt, ΔipaH7.8 and ΔipaH9.8 successfully expanding within the enteroid/colonoid epithelium. Scale bars: 50 µm. (B) Quantification of the GFP-positive area confirms successful intraepithelial expansion of all strains. Data is plotted as mean + SD of 5-6 (enteroids) or 4-5 (colonoids) replicates per strain, with one replicate corresponding to the mean at least 3 (enteroids) or 2 (colonoids) fields of view in an individually infected well.

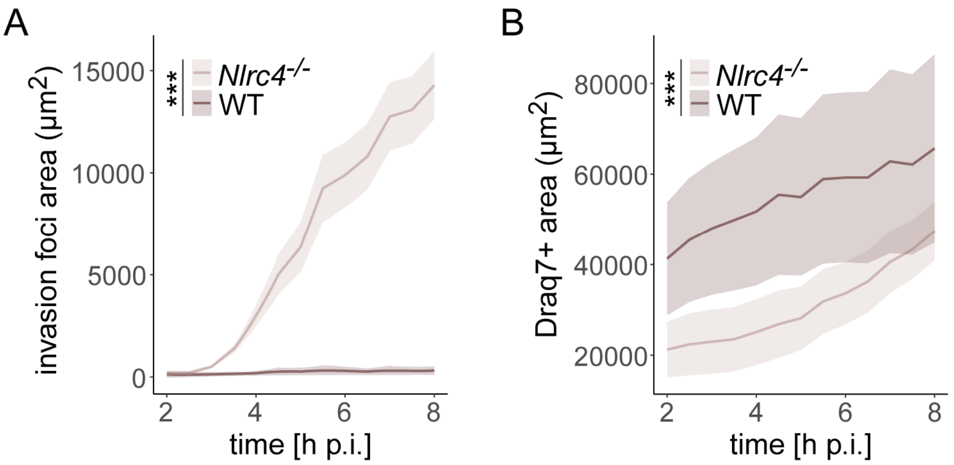

Supplementary Figure S8. *Nlrc4*-deficient, but not WT murine enteroid-derived monolayers are permissive for *Shigella* colonization. (A-B) WT and *Nlrc4*$^{-/-}$ murine enteroid-derived monolayers were infected with *Shigella* wt p*uhpT*-GFP (MOI 200 + centrifugation) and stained with Draq7. (A) Quantification of the GFP-positive area indicates that only *Nlrc4*$^{-/-}$ monolayers are permissive for *Shigella* invasion and subsequent intraepithelial expansion. (B) Quantification of the Draq7-positive area reveals reduced cell death in infected *Nlrc4*$^{-/-}$ monolayers. Data is plotted as mean + SD of 3-4 replicates per genotype, with one replicate corresponding to the mean of 3-4 fields of view for an individually infected well. Statistical significance was determined by two-way ANOVA and significance for the factor 'genotype' is indicated. ***, P<0.001.

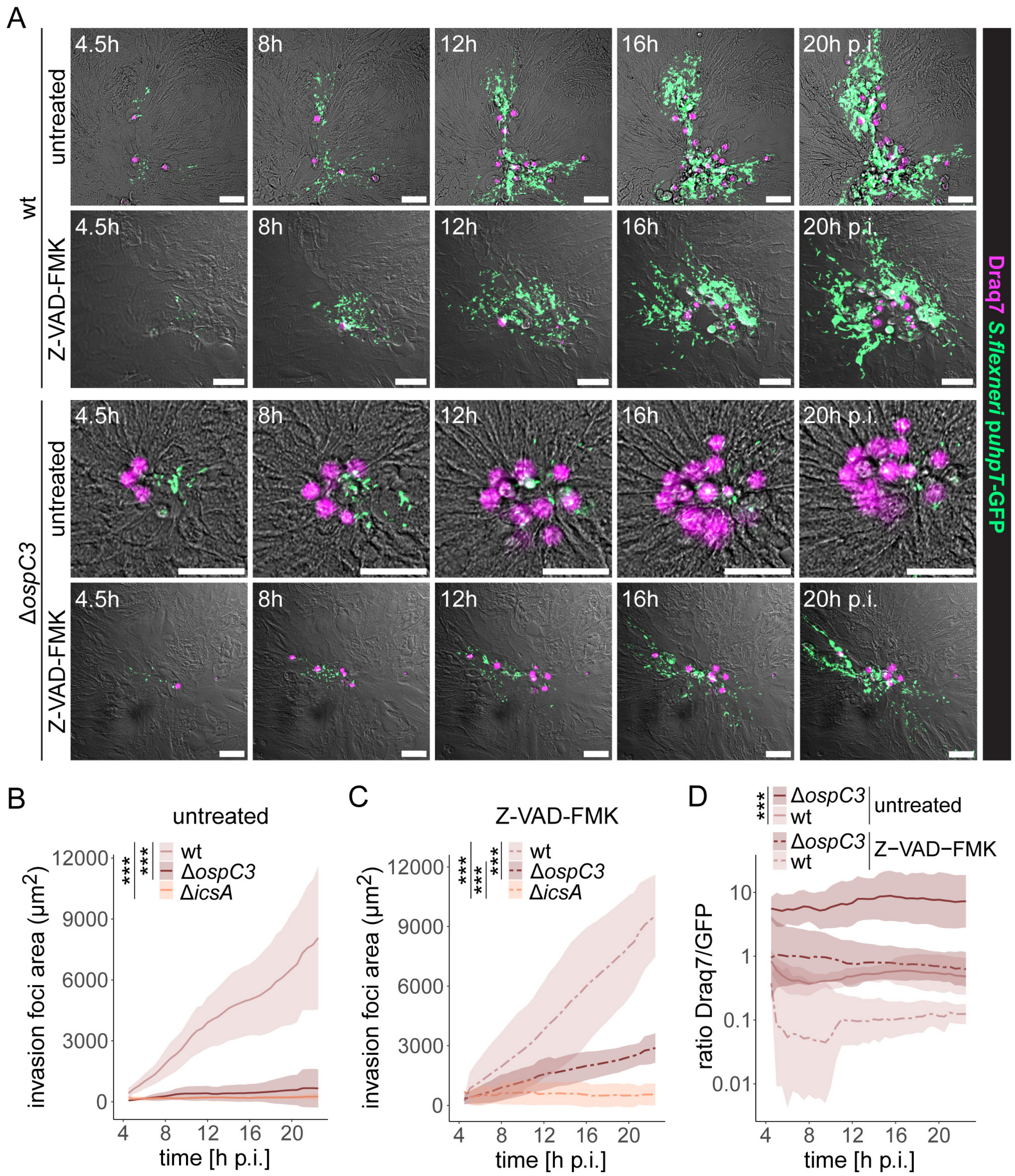

Supplementary Figure S9. OspC3 inhibits caspase-dependent cell death to allow for expansion of the *Shigella* population within the colonoid epithelium. (A-D) Colonoid-derived monolayers were infected with the indicated *Shigella* p*uhpT*-GFP strains as in Figure 5 in the presence or absence of the broad-spectrum caspase inhibitor Z-VAD-FMK and individual invasion foci were followed by time-lapse microscopy. (A) Representative images of Z-VAD-FMK treatment restoring *Shigella* Δ*ospC3* intraepithelial expansion to wt levels. Scale bars: 50µm. Quantification of the GFP-positive area in (B) untreated and (C) Z-VAD-FMK treated monolayers over time. (D) The Draq7-to-GFP ratio for *Shigella* Δ*ospC3* was reduced to wt levels upon Z-VAD-FMK treatment. Data is plotted as mean + SD of 4-5 replicates per strain and treatment (2 replicates for Δ*icsA*, Z-VAD-FMK), with one replicate corresponding to the mean of 2-8 foci in an individually infected well. Statistical significance was

determined by two-way ANOVA with Tukey's HSD post hoc test. Significance for comparisons among all strains for the respective treatment is indicated. ***, P<0.001.

SUPPLEMENTARY MOVIES

Supplementary Movie S1. *Salmonella* Δ*fljBfliC* but not *Shigella* wt stably adheres to the epithelium at the enteroid bottom plane. Exemplary time-lapse movies of *Salmonella* Δ*fljBfliC* (top panels) and *Shigella* wt (bottom panels) movements directly after microinjection (0 min p.i.; left panels) and at 60-180 min p.i. (right panels). Arrowheads indicated non-moving, adherent bacteria. Enteroids were microinjected with *Salmonella* Δ*fljBfliC* and *Shigella* wt p*rpsM*-mCherry as described in Figure 2. Frame rate: 500 ms; scale bars: 20 µm.

Supplementary Movie S2. *Salmonella* Δ*fljBfliC* but not *Shigella* wt stably adheres to the side epithelium of microinjected enteroids. Exemplary z-stacks of enteroids microinjected with *Salmonella* Δ*fljBfliC* (top panels) and *Shigella* wt (bottom panels) as described in Figure 2. Overlays of DIC and fluorescent (p*rpsM*-mCherry; red) channels directly after microinjection (0 min p.i.; left panels) and at 60 min p.i. (bottom panels) are shown. Distance between slices: 2-5 µm; scale bars: 50 µm.

Supplementary Movie S3. *Shigella* wt but not Δ*ospC3* or Δ*icsA* expand and spread efficiently within enteroid-derived monolayers. Exemplary time-lapse movies of enteroid-derived monolayers infected with *Shigella* wt (left), Δ*ospC3* (middle) or Δ*icsA* (right) as described in Figure 4. Overlays of DIC and fluorescent (p*uhpT*-GFP; green and Draq7; magenta) channels are shown. Time is displayed as hours:min. Scale bars: 20 µm.

SUPPLEMENTARY TABLES

Table S1. Strains and plasmids used in this study.

**Strain**	**Genotype**	**Reference**
*S.*Tm wt	SL1344, wt (SB300, Sm^{R})	(1)
*S.*Tm Δ*invG*	SL1344, Δ*invG* (SB161, Sm^{R})	(2)
*S.*Tm Δ*SPI-4*	SL1344, Δ*SPI-4* (Sm^{R}, Kan^{R})	(3)
*S.*Tm Δ*fljBfliC*	SL1344, Δ*fljBfliC* (Sm^{R}, Kan^{R})	(4)
*S.*Tm Δ*fljBfliC* Δ*fimH*	SL1344, Δ*fljBfliC* Δ*fimH* (Sm^{R}, Kan^{R})	This study
*S.*Tm Δ*fljBfliC* Δ*SPI-4*	SL1344, Δ*fljBfliC* Δ*SPI-4* (Sm^{R}, Kan^{R})	This study
*S.*Tm Δ*fljBfliC* Δ*invG*	SL1344, Δ*fljBfliC* Δ*invG* (Sm^{R}, Kan^{R})	This study
*S.*Tm Δ*motA* Δ*SPI-4*	SL1344, Δ*motA* Δ*SPI-4* (Sm^{R}, Kan^{R})	This study
S. flexneri wt	M90T, wt	(5)
S. flexneri Δ*mxiD*	M90T, Δ*mxiD* (Kan^{R})	(6)
S. flexneri Δ*ospC3*	M90T, Δ*ospC3* (Kan^{R})	This study
S. flexneri Δ*icsA*	M90T, Δ*icsA* (Kan^{R})	This study
S. flexneri Δ*ipaH7.8*	M90T, Δ*ipaH7.8* (Kan^{R})	This study
S. flexneri Δ*ipaH9.8*	M90T, Δ*ipaH9.8* (Kan^{R})	This study
**Plasmid**	**Description**	**Reference**
pFPV-mCherry	p*rpsM*-mCherry (constitutive)	(7)
pM975	p*ssaG*-GFP (SPI2-dependent, vacuolar)	(8, 9)
p*uhpT*-GFP	p*uhpT*-GFP (cytosolic)	Related to p*uhpT*-mCherry in (10)

Table S2. Primers used in this study.

**Primer**	**Sequence (5'-3')**
k1	CAGTCATAGCCGAATAGCCT
k2	CGGTGCCCTGAATGAACTGC
fimH-scr-fwd	CCAGTATGGTCGGCGATGAT
fimH-scr-rev	GGTTAACCGACTTGTTACTG
SPI4-scr-fwd	CGGTAGAGAATGGTCGGTAT
SPI4-scr-rev	GTGCTGACCTGATACGCTAT
invG-scr-fwd	CATGGTTACTCATCGCCTTC
invG-scr-rev	CATCCTGATGAGATGTCTGC
ospC3-del-fwd	ATAATGTTATCTAAATAACCACAGATAAAAACGCACATAATTATTCCGGGGATCCGTCGACC
ospC3-del-rev	CTTCGATAATCGACGACATTATTATTTGGCCGAGCTTTTAGGGTGTAGGCTGGAGCTGCTTC
icsA-del-fwd	ACGGAATCTTTTCAGGGGTTTATCAACCACTTACTGATAATAATTCCGGGGATCCGTCGACC
icsA-del-rev	GTCCCAGAGAAATGCAGGACATCAACACGCCCTGCATTTTTAGTGTAGGCTGGAGCTGCTTC
ipaH7.8-del-fwd	AAATATTTATTCTCACAAATATAAGGTTGACCTAGCATTATGATTCCGGGGATCCGTCGACC
ipaH7.8-del-rev	GGTCACAGTTTTTCCGGAGTCAATCCGGTCTGCGGTTTATGCGTGTAGGCTGGAGCTGCTTC
ipaH9.8-del-fwd	TGAAACAGTATCGTTTTTTTACAGCCAATTTTGTTTATCCTTATTCCGGGGATCCGTCGACC
ipaH9.8-del-rev	AGGTTTCACCTGTCCTATCACTGGCGCTGACAGTTTTATGCGGTGTAGGCTGGAGCTGCTTC
ospC3-scr-fwd	CATACAAGAAAGTGTCCAGT
ospC3-scr-rev	GGAAAGAGGGACCATCTCCA
icsA-scr-fwd	GGTAAATTTCTCCCGTTGCA
icsA-scr-rev	GGCATACCATCATGTGCACA
ipaH7.8-scr-fwd	CTTGCTCACTGGAATGTTGA
ipaH7.8-scr-rev	CCCTCAAAAAAGTCAGTTGC
ipaH9.8-scr-fwd	GAACAATACGGTGCAAACAG
ipaH9.8-scr-rev	CGTAAGCCGGTACGTATTGA

scr, screening primer; del, deletion primer; fwd, forward primer; rev, reverse primer

References for Supplementary Material

1. Hoiseth SK, Stocker BAD. 1981. Aromatic-dependent Salmonella typhimurium are non-virulent and effective as live vaccines. Nature 291:238–239.

2. Kaniga K, Bossio JC, Galán JE. 1994. The Salmonella typhimurium invasion genes invF and invG encode homologues of the AraC and PulD family of proteins. Mol Microbiol 13:555–568.

3. Gerlach RG, Jäckel D, Stecher B, Wagner C, Lupas A, Hardt W-D, Hensel M. 2007. Salmonella Pathogenicity Island 4 encodes a giant non-fimbrial adhesin and the cognate type 1 secretion system. Cell Microbiol 9:1834–1850.

4. Samperio Ventayol P, Geiser P, Di Martino ML, Florbrant A, Fattinger SA, Walder N, Sima E, Shao F, Gekara NO, Sundbom M, Hardt W-D, Webb D-L, Hellström PM, Eriksson J, Sellin ME. 2021. Bacterial detection by NAIP/NLRC4 elicits prompt contractions of intestinal epithelial cell layers. Proc Natl Acad Sci U S A 118:e2013963118.

5. Sansonnetti PJ, Kopecko DJ, Formal SB. 1982. Involvement of a plasmid in the invasive ability of Shigella flexneri. Infect Immun 35:852–860.

6. Skovajsová E, Colonna B, Prosseda G, Sellin ME, Di Martino ML. 2022. The VirF21:VirF30 protein ratio is affected by temperature and impacts Shigella flexneri host cell invasion. FEMS Microbiol Lett 369.

7. Drecktrah D, Levine-Wilkinson S, Dam T, Winfree S, Knodler LA, Schroer TA, Steele-Mortimer O. 2008. Dynamic behavior of Salmonella-induced membrane tubules in epithelial cells. Traffic 9:2117–2129.

8. Sellin ME, Müller AA, Felmy B, Dolowschiak T, Diard M, Tardivel A, Maslowski KM, Hardt W-D. 2014. Epithelium-intrinsic NAIP/NLRC4 inflammasome drives infected enterocyte expulsion to restrict Salmonella replication in the intestinal mucosa. Cell Host Microbe 16:237–248.

9. Hapfelmeier S, Stecher B, Barthel M, Kremer M, Müller AJ, Heikenwalder M, Stallmach T, Hensel M, Pfeffer K, Akira S, Hardt W-D. 2005. The Salmonella pathogenicity island (SPI)-2 and SPI-1 type III secretion systems allow Salmonella serovar typhimurium to trigger colitis via MyD88-dependent and MyD88-independent mechanisms. J Immunol 174:1675–1685.

10. Hausmann A, Böck D, Geiser P, Berthold DL, Fattinger SA, Furter M, Bouman JA, Barthel-Scherrer M, Lang CM, Bakkeren E, Kolinko I, Diard M, Bumann D, Slack E, Regoes RR, Pilhofer M, Sellin ME, Hardt W-D. 2020. Intestinal epithelial NAIP/NLRC4 restricts systemic dissemination of the adapted pathogen Salmonella Typhimurium due to site-specific bacterial PAMP expression. Mucosal Immunol 13:530–544.
